# Regulatory Effect of DNA Topoisomerase I on T3SS Activity, Antibiotic Susceptibility and Quorum- Sensing-Independent Pyocyanin Synthesis in *Pseudomonas aeruginosa*

**DOI:** 10.3390/ijms20051116

**Published:** 2019-03-05

**Authors:** Rong Yan, Shikun Hu, Ning Ma, Peiqing Song, Qingqing Liang, Huiqun Zhang, Yanqi Li, Lixin Shen, Kangmin Duan, Lin Chen

**Affiliations:** 1 Key Laboratory of Resources Biology and Biotechnology in Western China, Ministry of Education, College of Life Sciences, Northwest University, Xi’an 710069, Shaanxi, China; yanrongswallow@gmail.com (R.Y.); hushikun@stumail.nwu.edu.cn (S.H.); maning@stumail.nwu.edu.cn (N.M.); 201631710@stumail.nwu.edu.cn (P.S.); qingqingl@stumail.nwu.edu.cn (Q.L.); baihezhaohui@stumail.nwu.edu.cn (H.Z.); shenlx@nwu.edu.cn (L.S.); 2 Department of Oral Biology, Rady Faculty of Health Sciences, University of Manitoba, 780 Bannatyne Ave., Winnipeg, MB R3E 0W2, Canada; liy34544@myumanitoba.ca; 3 Department of Medical Microbiology and Infectious Diseases, Rady Faculty of Health Sciences, University of Manitoba, 780 Bannatyne Ave, Winnipeg, MB R3E 0W2, Canada

**Keywords:** DNA Topoisomerase, Pyocyanin, *Pseudomonas aeruginosa*, *prtN*, type III secretion system, antibiotic susceptibility, biofilm

## Abstract

Topoisomerases are required for alleviating supercoiling of DNA during transcription and replication. Recent evidence suggests that supercoiling of bacterial DNA can affect bacterial pathogenicity. To understand the potential regulatory role of a topoisomerase I (TopA) in *Pseudomonas aeruginosa,* we investigated a previously isolated *topA* mutation using genetic approaches. We here report the effects of the altered topoisomerase in *P. aeruginosa* on type III secretion system, antibiotic susceptibility, biofilm initiation, and pyocyanin production. We found that *topA* was essential in *P. aeruginosa*, but a transposon mutant lacking the 13 amino acid residues at the C-terminal of the TopA and a mutant, named *topA*-RM, in which *topA* was split into three fragments were viable. The reduced T3SS expression in *topA*-RM seemed to be directly related to TopA functionality, but not to DNA supercoiling. The drastically increased pyocyanin production in the mutant was a result of up-regulation of the pyocyanin related genes, and the regulation was mediated through the transcriptional regulator PrtN, which is known to regulate bacteriocin. The well-established regulatory pathway, quorum sensing, was unexpectedly not involved in the increased pyocyanin synthesis. Our results demonstrated the unique roles of TopA in T3SS activity, antibiotic susceptibility, initial biofilm formation, and secondary metabolite production, and revealed previously unknown regulatory pathways.

## 1. Introduction

Topoisomerases are enzymes found in all three domains of life, which control the topology of DNA in all cells [1]. Topoisomerase Ι activity is required for preventing hyper-negative supercoiling of DNA during transcription [2], and along with gyrase activity, directly influences the chromosome supercoiling. The level of DNA supercoiling is controlled by balanced actions of these two opposing enzymes [3]. Mounting evidence strongly suggests that various topological changes in DNA (DNA supercoiling) are a fundamental regulatory principle in the control of bacterial gene expression, enabling bacteria to adapt to environmental changes including antibiotic challenges, host defenses, and competition from neighboring microorganisms [3,4,5,6,7]. It is also known that environmental cues could influence virulence factors, biofilm, and motility by modulating the supercoiling of bacterial DNA [2,3,4,5,8,9]. The discovery of compounds that enhance DNA cleavage by type I topoisomerase has made topoisomerase a potential antibacterial target [1,10,11,12]. 

*Pseudomonas aeruginosa* (*P. aeruginosa*) is a ubiquitous Gram-negative bacterium present in various environments including water and soil. It is also a major opportunistic pathogen that causes diverse infections in humans, causing devastating diseases such as chronic lung infections, burn wound infections, urinary tract infections, and implant or biomaterial associated infections [13]. Antibiotic resistant *P. aeruginosa* poses a significant challenge to human health [14]. In spite of the importance of topoisomerases, the topoisomerase I in *P. aeruginosa* has not been fully investigated. In contrast to *Escherichia coli* (*E. coli*), *P. aeruginosa* possesses only one gene encoding a type IA topoisomerase, TopA [15]. Its relationship with cell viability or other phenotypical characteristics has not yet been revealed. 

*P. aeruginosa* is able to produce a number of phenazine compounds that are redox active pigmented molecules involved in bacterial competition and pathogenicity. As an extracellular virulence factor, the major phenazine compound pyocyanin (PYO) produced by *P. aeruginosa* binds directly to DNA to promote biofilm formation [16]. PYO production is essential for the success of both acute and chronic lung infection in mice, indicating that this compound plays a crucial role in host-pathogen interaction [17]. Besides its major function as a virulence factor and electron transfer facilitator [18], PYO also serves as a signaling molecule in *P. aeruginosa*, controlling a limited set of genes during a stationary growth phase [19]. In *P. aeruginosa*, two copies of the seven-gene operon *phz1* (*phzA1B1C1D1E1F1G1*) and *phz2* (*phzA2B2C2D2E2F2G2*) are known to be responsible for the biosynthesis of phenazine compounds. In addition, the products encoded by *phzM* and *phzS* are required to convert the intermediate phenazine-1-carboxylic acid (PCA) to the final products such as 1-hydroxphenazine and PYO [20]. The expression of phenazine synthesis genes are controlled by complex regulation networks and responds to environmental cues. The recognized regulatory systems include two component systems, quorum sensing (QS) systems, sRNAs, and environmental cues [21,22,23,24]. Production of phenazine compounds is regulated by the two acyl-homoserine lactone mediated *las* and *rhl* systems and the *Pseudomonas* quinolone signal mediated system (PQS). Both the PQS and *rhl* system are required for phenazine production [24,25,26]. However, in many conditions, the precise molecular cues that interact with regulatory proteins to control phenazine production remain to be understood completely. 

As the means for survival in diverse environmental conditions and to interact with the members of its residing microbial communities or the host, *P. aeruginosa* also has the ability to form biofilms and possesses specialized protein secretion systems. During *P. aeruginosa* infection, the transition to chronic infection is often accompanied by the formation of biofilm communities while the contact-dependent type III secretion system (T3SS) is required for acute infection [27,28,29]. The formation of biofilms is the main cause of the difficulty in eradicating *P. aeruginosa* chronic infections [30].

In this report, we present evidence that demonstrates that topoisomerase I was essential for bacterial viability in *P. aeruginosa* and altered topoisomerase I activity influences important bacterial activities, including T3SS and antibiotic susceptibility. The serendipitous observation of elevated PYO synthesis in the *topA* transposon insertion mutant and the *topA*-RM mutant constructed led to the discovery of a QS-independent regulatory pathway of PYO production in *P. aeruginosa* where PrtN, which induces the production of the bacteriocin pyocin, plays an indispensable role in PYO over-production in the *topA* mutant background. The results that link the DNA topoisomerase I with T3SS and antibiotic resistance with and without the involvement of DNA supercoiling have also been discussed. 

## 2. Results

### 2.1. TopA Is Essential for Cell Viability in P. aeruginosa

In our previous studies, we carried out a transposon mutagenesis to screen the genome of *P. aeruginosa* for genes that affect T3SS expression and one of the genes characterized was PA1611 [31]. Another gene disrupted by transposon that affected T3SS was PA3011 encoding DNA topoisomerases I (*topA*). Alongside the T3SS activity, another obvious change of the mutant was the dramatic increase of pigmentation of colonies on solid medium. PCR and sequencing analysis indicate that, in the transposon mutant, the transposon inserted into *topA* at the site of 2569 bp (total 2607 bp), eliminating 13 amino acid residues from the translated topoisomerase I. 

**Figure 1:**
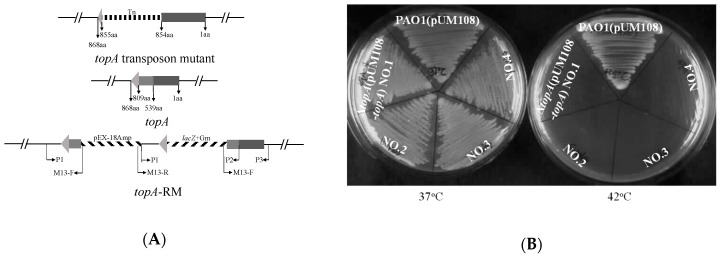
Schematic depiction of the genomic organization of *topA* in *topA*-RM and *topA* transposon mutant, and growth test showing the essentiality of TopA. (**A**) Schematic presentation of the genetic structure of *topA* in *topA*-RM and *topA* transposon mutant. Top: *topA* was separated by the transposon element (Tn). Middle: intact *topA* gene on the chromosome. The amino acid residues at which split-up occurred during the recombination event are indicated. Bottom: the three *topA* fragments in *topA*-RM are shown. P1, P2, P3, are PCR primers with their binding sites shown. M13-F and M13-R are universal primers that bind to the pEX18Amp plasmid. These primers were used to determine the genetic structure of *top*-RM (Table S1, Figure S1). (**B**) Growth of the *topA* chromosomal deletion mutant that carried a copy of *topA* on the temperature-sensitive plasmid pUM108 at 37 °C, whereas there was no growth at 42 °C. PAO containing pUM108 was able to grow at both temperatures. Four different isolates (No.1 to 4) of the *topA* conditional mutant are shown.

To further characterize this mutant, construction of a knockout mutant was attempted multiple times using a plasmid-based homologous combination approach [32], but failed to attain, suggesting that, unlike the *topA* in *Escherichia coli* [33], *topA* in *P. aeruginosa* is essential for viability. However, when we tried to construct the insertion mutant with *topA* disrupted at the site similar to the transposon, we were able to obtain an intermediate construct (named *topA*-RM) which gave the same phenotypical changes as the transposon mutant. The genotypic configuration of *topA*-RM was determined by DNA sequencing (data not shown) and PCR analysis (Figure S1). It contains three *topA* fragments separated by the plasmid DNA and *lacZ*:Gm cassette, as shown in Figure 1A. Apparently, either the truncated *topA* alone (lacking 59 amino acid residues at the C-terminus) or these fragments together could function to sustain the viability of the cells. As the transposon mutant has a truncated TopA lacking the C-terminal 13 residues was viable at our culture condition, it is more likely that the TopA without the C-terminal residues could sustain viability in *P. aeruginosa*. The growth of *topA*-RM and the transposon mutant was the same as that of PAO1 in LB (Figure S2). 

To confirm that the *topA* is essential for *P. aeruginosa* viability, a temperature-sensitive plasmid pUM108, which cannot replicate at 42 °C [34], was used to construct a conditional knock-out strain. The plasmid pUM108-*topA* containing the whole *topA* and its promoter was introduced into the strain before the *topA* was deleted. The resultant strain with *topA* deleted from the chromosome was selected on medium supplemented with sucrose and carbenicillin at 37 °C, and confirmed by PCR analysis using the primers (P0 and *topA*-up). The primer *topA*-up binds the chromosome of PAO1, but not the complementation plasmid. As shown in Figure 1B, the *topA* conditional mutant could grow at 37 °C when pUM108-*topA* was present in the cell, but not at 42 °C when pUM108-*topA* was unable to replicate. These results confirm that TopA (topoisomerase I) was required for *P. aeruginosa* viability. 

It is clear that, while TopA is essential in *P. aeruginosa*, the truncated topoisomerase I retained partial activity that was sufficient for viability under the experimental conditions. The amino acid residues at the C-terminus of TopA apears to be dispensable for viability, yet their absence partially impaired the function of TopA. 

### 2.2. Impaired TopA Has Pleotropic Effects on T3SS Expression, Phenazine Production, Antibiotic Susceptibility and Biofilm Formation

**Figure 2:**
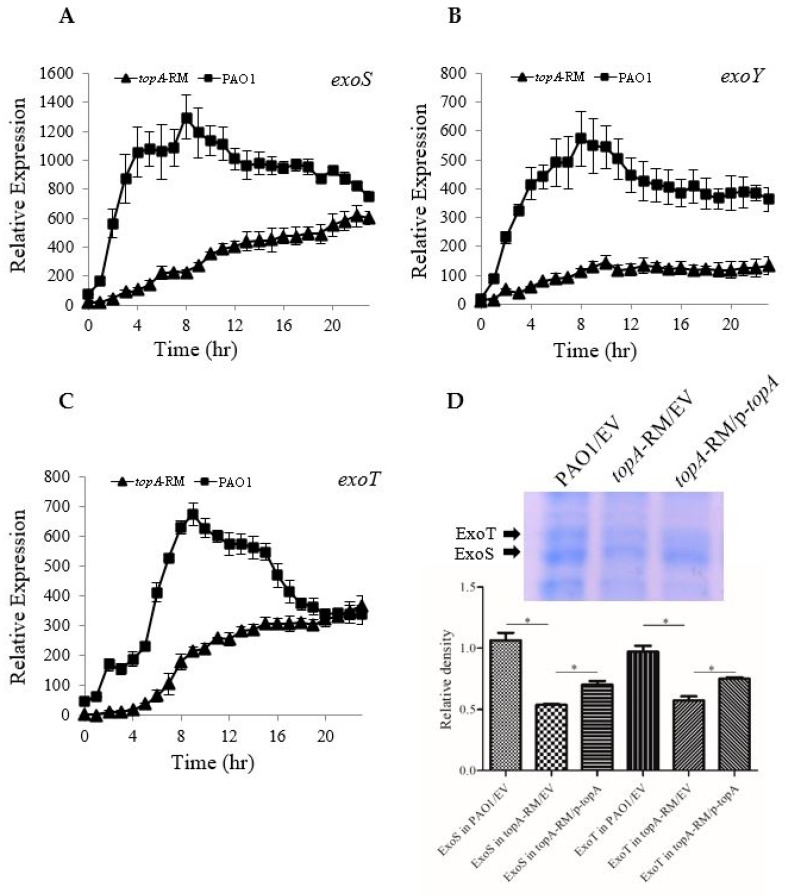
Reduced expression of T3SS effectors in *topA*-RM. (**A–C**) The expression profiles of T3SS effectors measured using p-*lux* promoter-reporter system. The values are presented as cps normalized to OD_595_. The values of *topA*-RM are shown as triangles and those in PAO1 as squares (data shown as averages from triplicate experiments ± standard errors of the means). (**D**) Effect of altered TopA on T3SS effectors protein secretion. A representative gel image is shown on top and the quantitative values (mean and SD) of ExoS and ExoT calculated from the results of three repeats are shown underneath. Proteins in the culture supernatants of various strains grown in T3SS-inducing condition were precipitated by TCA and analyzed by SDS-PAGE, followed by staining with Coomassie blue. The relative quantification of the secreted ExoS protein on the gel was evaluated by the relative density of the bands using the GeneTools software (Syngene, Cambridge, United Kingdom). Two-tailed unpaired *t*-test was performed using GraphPad software version 5.0 (*, *P* < 0.05). ExoT and ExoS are indicated by solid arrows, as established in previous reports [28,31]. EV: empty vector as a control. p-*topA*: *topA* complementation construct.

Using the mutant, *topA*-RM, the effect of altered *topA* on T3SS and phenazine production was verified. In agreement with our previous observations with the transposon mutant, *topA*-RM showed a significant decrease in T3SS effector *exoS* promoter activity (Figure 2A) and a significant increase in pigmentation production (Figure S3). *exoS* encodes exoenzyme S, an ADP-ribosyltransferase. Similarly, the promoter activities of both *exoT* and *exoY*, which encoded two other T3SS effectors, a promiscuous cyclase and a GTPase, were also down-regulated (Figure 2B,C). To confirm the effect on T3SS, we also compared the secreted T3SS effectors production in the mutant and the wild type PAO1. As shown in Figure 2D, less T3SS effectors ExoT and ExoS were present in the mutant culture. 

**Figure 3:**
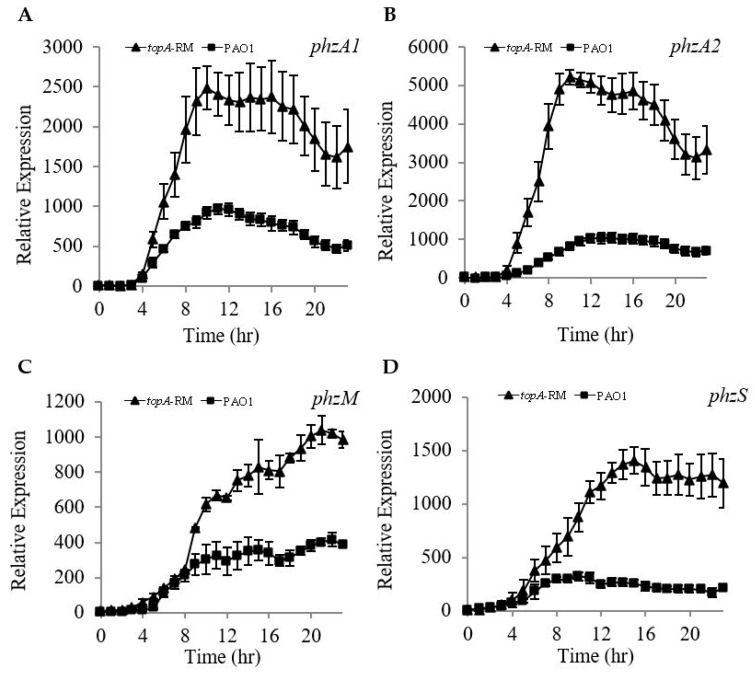
Activated promoter activity of the genes involved in PYO synthesis. The promoter activity of *phzA1* operon (**A**), *phzA2* operon (**B**), *phzM* (**C**), and *phzS* (**D**) in *topA*-RM was measured using the p-*lux* reporter system. The expression levels are presented as cps normalized to OD_595_. The values in *topA*-RM are shown as triangles and those in PAO1 as squares (data shown as averages from triplicate experiments ± standard errors of the means).

Noticing the apparent green color of *topA*-RM cultures (Figure S3), we suspected that *topA*-RM produced more PYO which is green in oxidized form. In *P. aeruginosa* PAO1, the production of PYO is carried out by enzymes encoded by genes in the *phzA1* and *phA2* operons together with *phzS* and *phzM* [20]. Genes in the homologous 7-genes operon *phzA1* and *phzA2* are required to synthesize the intermediate molecule, phenazine-1-carboxylic acid (PCA), which is then converted to PYO by PhzM and PhzS. Therefore, the promoter activity of PYO related genes (including *phzA1*, *phzA2*, *phzM*, and *phzS*) was monitored. As shown in Figure 3A–D, all of these genes were up-regulated in *topA*-RM. To confirm the increased production of PYO in the mutant, we quantified PYO production using HPLC analysis in *topA*-RM, PAO1 and complementation strains. The results indicate that *topA*-RM produced much more PYO than the wild type (Figure S4). The complementation of *topA* in the strain with the p-*topA* plasmid could restore PYO production to the wild-type levels. The results indicate that the impaired *topA* caused increased PYO production. 

**Figure 4:**
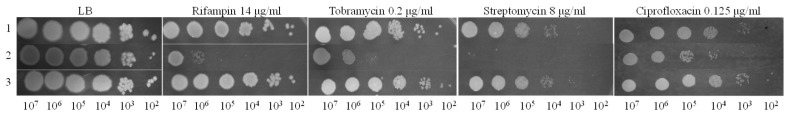
Comparison of antibiotic susceptibilities among PAO1, *topA*-RM and the complementation strain. The growths of the three strains on different antibiotics are shown. Row 1, PAO1 with pUCP26 (the empty plasmid); row 2, *topA*-RM with empty vector pUCP26. The colonies appear darker due to PYO over-production; row 3, *topA*-RM complemented by an intact *topA* carried on pUCP26. Serial dilutions are shown underneath. The LB plate on the left contained no antibiotics.

It has been reported that the reduction of topoisomerase I leads to the inefficient transcription of several genes, conferring various pleiotropic effects in *Mycobacterium smegmatis* [35]. To test potential pleiotropic effect of altered TopA in *P. aeruginosa*, selected phenotypes relevant to its survival in adversary conditions and pathogenicity were examined in *topA*-RM. As shown in Figure 4, impaired *topA* made the bacterium more susceptibility to the following antibiotics: Ciprofloxacin, rifampin, streptomycin, and tobramycin. No change was observed with other antibiotics tested, including chloramphenicol, piperacillin, erythromycin, meropenem, spectinomycin, cefotaxime, mitomycin, novobiocin, and roxithromycin (data not shown). Complementation of *topA*-RM with a plasmid carrying an intact copy of *topA* restored all the phenotypes to the levels compared to wild-type (Figure 4).

**Figure 5:**
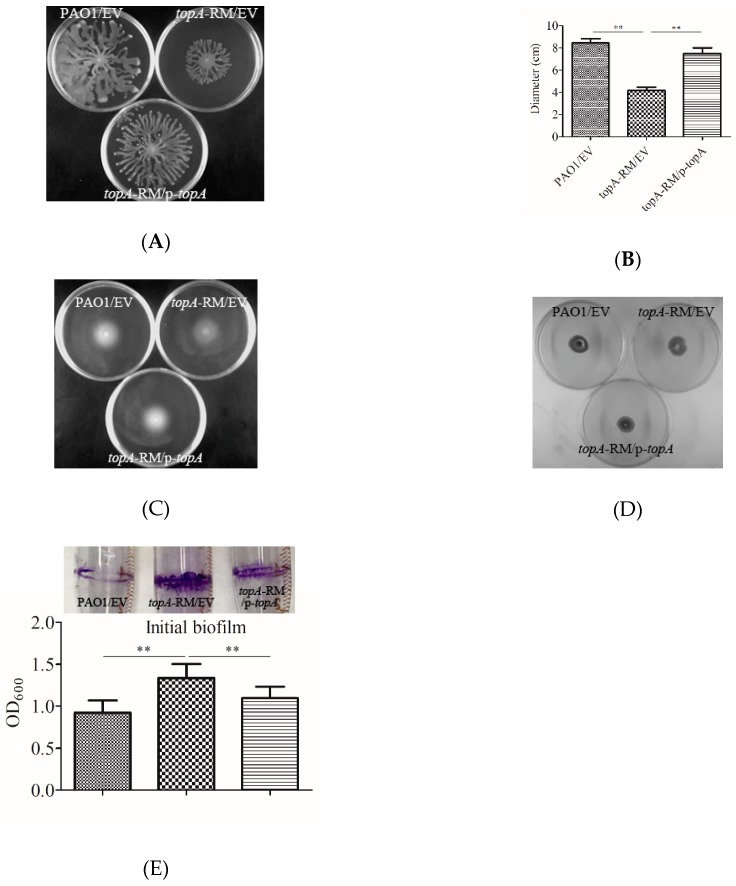
Changed motility and initial biofilm formation in *topA*-RM. (**A**) Effect of altered TopA on swarming, (**B**) quantification of swarming motility by the diameters of the covered areas, (**C**) swimming, and (**D**) twitching motility. Overnight cultures were spotted onto corresponding plates (2 μL aliquots) and the plates were incubated at 37 °C. The images were captured using a LAS-3000 imager. (**E**) A photo of the biofilms binding to the tubes and quantification of the biofilms by crystal violet staining. The biofilms were analyzed after 16 h growth. The two-tailed unpaired *t*-test was carried out using GraphPad software (** *P* < 0.005 compared to WT or complemented strains). EV: Empty vector control. p-*topA*: *topA* complementation construct.

In addition, mutation of *topA* resulted in reduced swarming motility while the swimming and twitching motility were unchanged (Figure 5A–D). Using borosilicate tube binding assays, the *topA*-RM exhibited more initial biofilm formation than either the wild type or the complemented strain (Figure 5E). The increased biofilm initiation in *topA*-RM is consistent with the decreased swarming motility observed in this mutant as there is a negative correlation between biofilm-formation and swarming motility. These results suggest TopA potentially plays a role in modulating *P. aeruginosa* pathogenicity and survival.

### 2.3. DNA Supercoiling Is Elevated as a Result of Impaired TopA

**Figure 6:**
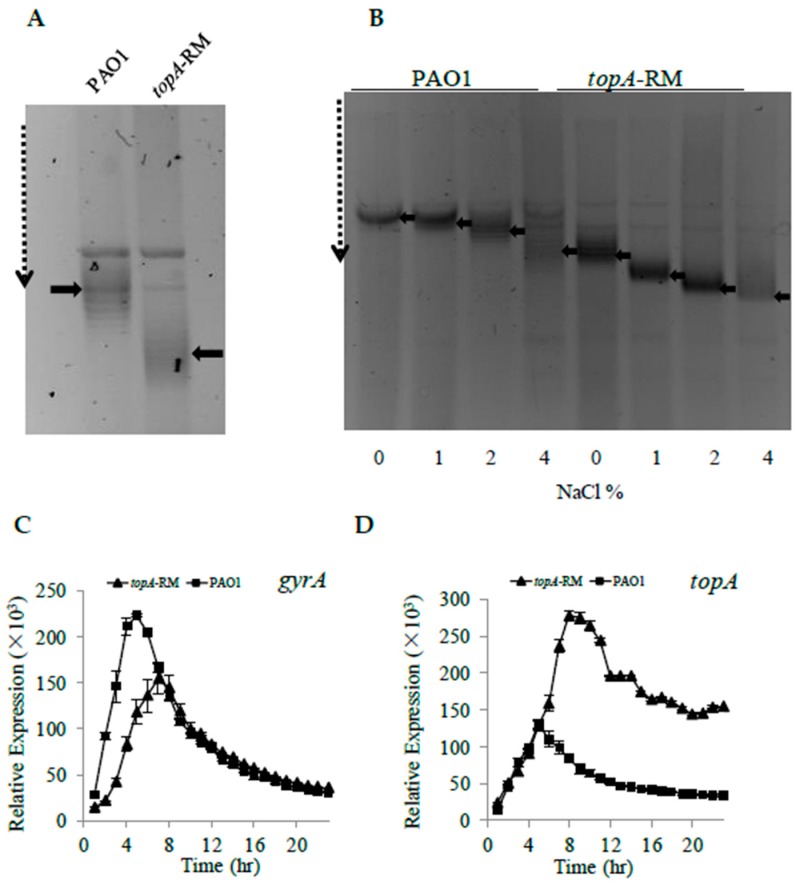
The DNA topological changes in *topA*-RM. (**A**) Higher DNA supercoiling level in *topA*-RM than in PAO1. The plasmid pUCP26 isolated from each strain was separated on 1% agarose gel containing 5 µg/mL chloroquine. Solid arrows indicate supercoiled plasmid; the dashed arrow points increased negative supercoiling levels. (**B**) Enhanced DNA supercoiling with increasing NaCl concentrations. Cells were treated at different concentration of NaCl for 3 h before plasmids were isolated. Topoisomers of plasmids from different treatments were analyzed by the chloroquine gel electrophoresis. (**C**,**D**) The expression profiles of *gyrA* (**C**) and *topA* (**D**) were measured using p-*lux* reporter system. The expression levels are presented as cps normalized to OD_595,_ and the values in *topA*-RM are shown as triangles and those in PAO1 as squares (data shown as averages from triplicate experiments ± standard errors of the means).

Protein sequence alignment showed that the TopA of *P. aeruginosa* shares considerable sequence identity (67.5%) with the Topo I of *E. coli* (Figure S5). DNA topoisomerases influence DNA supercoiling which in turn influences bacterial gene expression [4] and the topoisomerase I with truncated C-terminal domain in *E. coli* has lower affinity to DNA and affects antibiotic susceptibility [36,37]. The effect of the altered TopA on DNA supercoiling was examined in *topA*-RM by quantifying the distribution of topoisomers of the plasmid pUCP26 using agarose gel electrophoresis containing chloroquine [38]. When the plasmid topoisomers were separated on a gel containing 5 µg/mL chloroquine, the more negatively supercoiled topoisomers migrated further on the gel, whereas the more relaxed topoisomers were retained behind. The results revealed that the hyper-negative supercoiled form of the plasmid increased in the *topA*-RM compared with PAO1 (Figure 6A), indicating the impaired TopA caused a higher level of negative supercoiling. We also evaluated the topoisomers using a different plasmid (pKD-*exoY*) and observed similar result (Figure S6).

Environmental factors have the potential to modulate the supercoiling of the DNA [3,39,40], and high osmotic pressure, for instance, causes increased DNA supercoiling [7,41]. To further examine the DNA supercoiling levels in the *topA* partial mutant, plasmids DNA isolated from the cultures with NaCl at different concentrations were tested by chloroquine gel analysis. As shown in Figure 6B, negative supercoiling was markedly increased with elevated concentration of NaCl in both PAO1 and *topA*-RM. 

In bacteria, DNA supercoiling has a feedback effect on topoisomerase gene expression, which helps to maintain a homeostasis in supercoiling density of the genomic DNA [42,43]. In the simplest mechanism, increased negative DNA supercoiling inhibits the promoter activity of the genes that cause DNA negative supercoiling and DNA relaxation inhibits genes that cause DNA relaxation. To verify the increased negative supercoiling on the expression of topoisomerases, two reporter vectors named pKD-*topA* and pKD-*gyrA* were constructed. Consistence with the observation that DNA supercoiling was elevated in *topA*-RM, the expression of *topA* was upregulated and *gyrA* was downregulated in *topA*-RM (Figure 6C,D). 

### 2.4. Transcriptional Activity of T3SS and Production of Secreted Effectors Correlate with TopA Activity but not DNA Supercoiling

**Figure 7:**
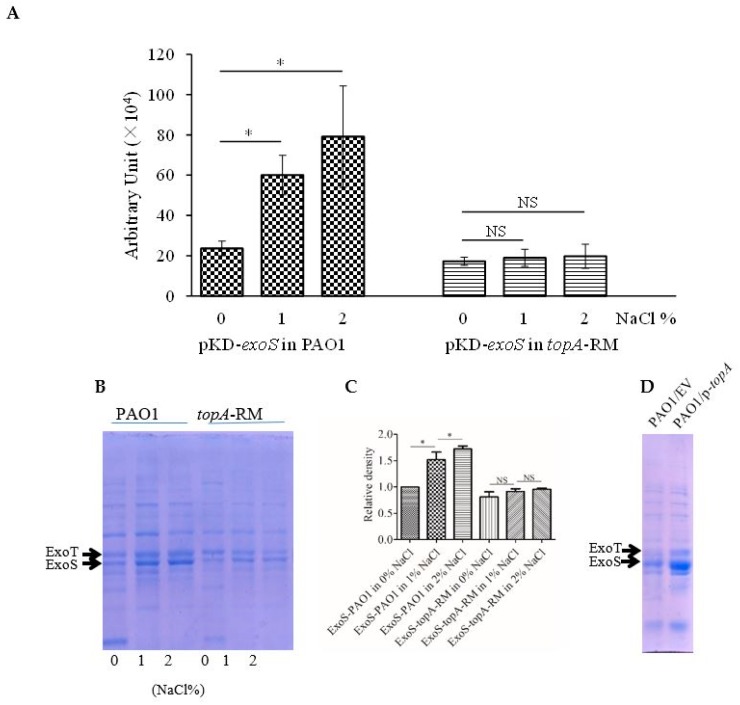
The correlation between T3SS effectors gene expression or effector secretion and the topoisomerase I, TopA. (**A**) The increased promoter activity of *exoS* with the increased concentration of NaCl in PAO1 but not in *topA*-RM. The reporter culture was serial diluted and spotted onto the plates with different concentrations of NaCl. After overnight incubation at 37 °C, light production of the reporter strain was recorded by a LAS-3000 imaging system (Fuji Corp.). The relative quantification of the promoter activities was evaluated by the software multi gauge version 3.0. The two-tailed unpaired *t*-test was used to analyze the data using GraphPad software version 5.0 (*, *P* < 0.05). NS: no significant difference. (**B**) Increased T3SS effectors protein secretion with elevated concentrations of NaCl in PAO1 but not in *topA*-RM. (**C**) Quantification of ExoS treated under different concentrations of NaCl. (**D**) Overexpression of *topA* on pUCP26 resulted in elevated T3SS effectors secretion. ExoT and ExoS bands are indicated by solid arrows, as established in previous reports [28,31]. EV: Empty vector as a control; p-*topA: topA* complementation construct. The results shown are representatives of three independent experiments with similar results.

Mounting evidences indicate that various topological changes in DNA including supercoiling serve as a fundamental regulatory principle in the control of bacterial gene expression [3,4,40]. To examine whether the elevated DNA supercoiling *topA*-RM was the cause of the decreased T3SS expression observed, both T3SS genes expression and T3SS effectors production were measured at different osmotic conditions. While it is known that increased osmotic pressure was caused by elevated NaCl concentrations induces increased DNA negative supercoiling [7] and the results above (Figure 6B) confirm that NaCl concentrations increased DNA supercoiling both in the wild type and the mutant, the expression of T3SS genes in the wild type, however, was up-regulated with elevated NaCl concentrations instead (Figure 7A). Similarly, enhanced production of T3SS effectors in the culture supernatant of PAO1 was observed when NaCl concentrations increased (Figure 7B). However, neither the expression of T3SS genes nor the secreted T3SS effectors was affected by the increased NaCl concentrations in *topA*-RM (Figure 7A,B). These results suggested that the increased negative supercoiling was not directly involved in the decreased production of T3SS effectors in *topA*-RM. Instead, a normal function of TopA seems to be required for T3SS. 

To verify whether TopA activity directly correlates with T3SS expression, complementation experiments were carried out using a plasmid carrying *topA*. The results indicate that a wild-type *topA* could restore T3SS secretion, but the mutant *topA* missing the 59 amino acid residues could not (Figure S7). In addition, the increased expression of *topA* in the wild type PAO1 carrying extra copies of *topA* on the plasmid induced the production of T3SS (Figure 7C,D), further supporting a potential positive correlation between TopA and T3SS expression.

**Figure 8:**
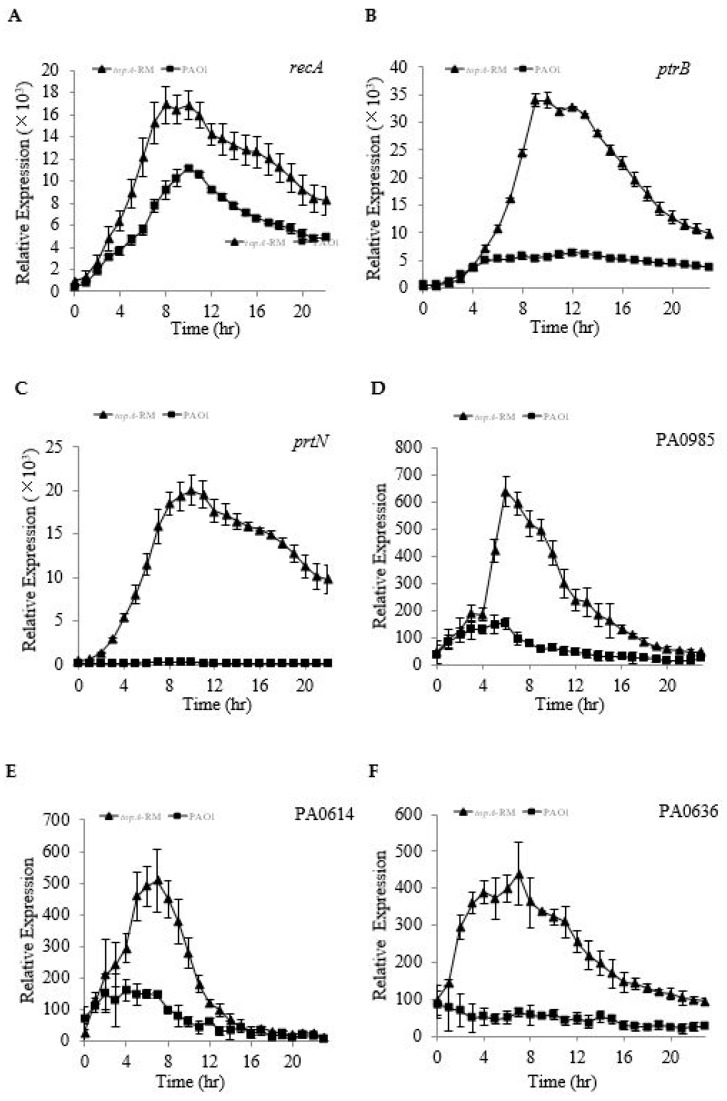
Comparison of expression profiles of the *recA* (**A**), *ptrB* (**B**), *prtN* (**C**)and pyocin related genes (**D**–**F**) in PAO1 and *topA*-RM. The expression levels are presented as cps normalized to OD_595_. The values in *topA*-RM are shown as triangles and those in PAO1 as squares (data are shown as averages from triplicate experiments ± standard errors of the means).

Inhibition of topoisomerase I activity by topoisomerase inhibitor seconeolitsine has been reported to up-regulate *recA* in *Streptococcus pneumoniae* [6]. In *P. aeruginosa*, it is known that the activation of RecA causes the cleavage of PrtR, which is the repressor of both PrtN and PtrB [44]. While PrtN is a transcriptional activator that activates pyocin biosynthetic genes, PtrB is a specific inhibitor of the T3SS. The de-repression of *ptrB* and *prtN* respectively suppresses the T3SS and activates the expression of genes involved in the synthesis of pyocin, a bacteriocin produced by *P. aeruginosa* [44]. Considering the possibility that the altered TopA in *topA*-RM could trigger an activation of *recA*, we examined the possibility that the repression of T3SS in *topA*-RM might be a result of the aberrant TopA in *topA*-RM causing the activation of *recA* and subsequent de-repression of *ptrB*. We hence compared the transcription levels of *recA*, *ptrB,* and *prtN* in *topA*-RM with those levels in PAO1. The result showed that the transcription activities of *recA*, *ptrB*, and *prtN* were all up-regulated in *topA*-RM (Figure 8A–C).

The transcriptional regulator *prtN* controls the production of bacteriocin pyocin in *P. aeruginosa*. To further examine the effect of impaired *topA* on PrtR and the PrtR-regulated genes, we measured the expression of pyocin synthase genes. As shown in Figure 8D–F, the genes involved in S- (PA0985), R- (PA0614), and F-type pyocin biosynthesis (PA0636) were all up-regulated in *topA*-RM. The results further support the effect of impaired *topA* on the PrtR-regulated genes.

**Figure 9:**
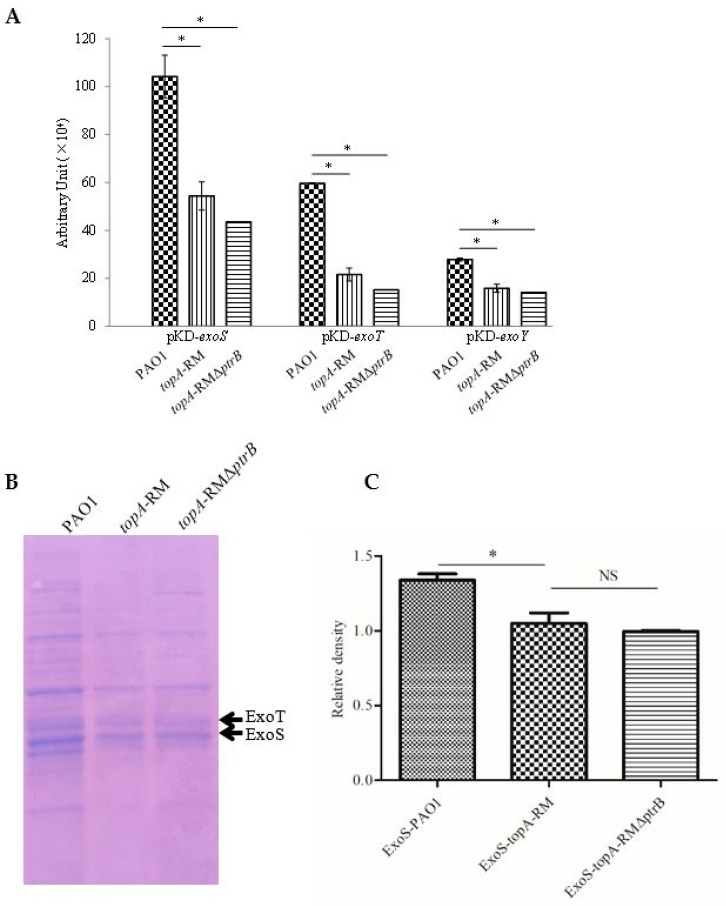
Comparison of T3SS genes expression and effectors production in PAO1, *topA*-RM and *topA*-RM*ΔptrB*. (**A**) T3SS effector genes expression in PAO1, *topA*-RM and *topA*-RM*ΔptrB*. The T3SS reporter strain cultures were serial diluted and spotted onto the LB plate. After overnight incubation at 37 °C, light production of the reporter strain was recorded by a LAS-3000 imaging system (Fuji Corp.). The relative quantification was evaluated by the software multi gauge version 3.0. (**B**) Secreted protein levels of T3SS effectors in PAO1, *topA*-RM and *topA*-RM*ΔptrB*. (**C**) Quatification of the ExoS production in PAO1, *topA*-RM and *topA*-RM*ΔptrB*. The proteins in the supernatant of different strain cultures were precipitated by TCA and analyzed by SDS-PAGE, followed by staining with Coomassie blue. ExoT (upper) and ExoS (lower) are indicated by solid arrows as established in previous reports [28,31]. The two–tailed unpaired *t*-test was used to analyze the data using the GraphPad software version 5.0 (*, *P* < 0.05). NS: No significant difference.

To verify whether the repression of T3SS in *topA*-RM (Figure 2) was a result of increased expression of T3SS negative regulator *ptrB*, a *ptrB* mutant was constructed in *topA*-RM. Both the expression of T3SS genes and secreted T3SS effectors proteins were tested in the *ptrB*-*topA* double mutant background. As shown in Figure 9A, no significant difference in T3SS expression was observed between *topA*-RM and the *ptrB*-*topA* double mutant. Comparison of T3SS effectors secretion also showed no obvious difference (Figure 9B,C). Apparently, the negative effect of the aberrant TopA on T3SS was not alleviated by the deletion of *ptrB*. Therefore, the repression of T3SS in *topA*-RM was not through the depression of *ptrB*, but more likely linked to the impaired TopA activity.

### 2.5. Elevated PYO Production Requires PrtN

**Figure 10:**
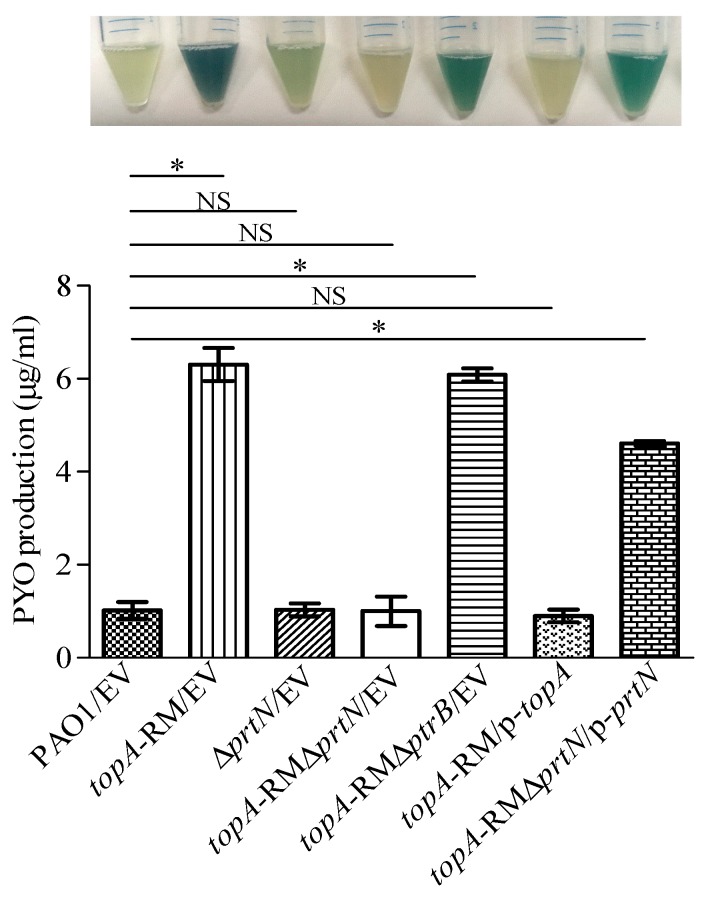
Comparison of the pyocyanin production in the wild-type PAO1 and mutant strains. Pyocyanin production was extracted from PB culture supernatants by chloroform and quantified using the optical density method (OD_520_) as described in materials and methods. EV: Empty vector in the strain. p-*topA* and p-*prtN* represent intact *topA* and *prtN* carried on pUCP26, respectively. The two-tailed unpaired *t*-test was used to analyze the data using GraphPad software version 5.0 (*, *P* < 0.05). NS: No significant difference. The means of values of three independent experiments are shown and the error bars indicate the standard deviations.

To investigate whether the increased production of PYO was due to the changes of PrtR in *topA*-RM, we deleted the PrtR-controlled *ptrB* and *prtN* in the *topA*-RM mutant background, respectively, and measured PYO production in the resultant mutant constructs *topA*-RM*ΔprtN* and *topA*-RM*ΔptrB*. As shown in Figure 10 (also Figure S4), although 4 to 5-fold more PYO was produced in *topA*-RM than PAO1, the deletion of *ptrB* did not affect PYO production in *topA*-RM whereas the deletion of *prtN* abolished the elevated production of PYO in *topA*-RM. The *topA*-RM*ΔprtN* strain produced similar amount of PYO as PAO1. The results suggest that *prtN* was involved in the induction of PYO by the altered TopA. In agreement with this conclusion, complementation of *prtN* on a plasmid partially restored the over-production of PYO in the *topA*-RM*ΔprtN* double mutant. However, deletion of *prtN* alone in the wild-type PAO1 did not affect PYO production (Figure 10 and Figure S4). Only in the *topA*-RM background was PrtN required for PYO production. In other words, the increased PYO production caused by impaired TopA depends on the de-repression of *prtN*.

### 2.6. Quorum Sensing Is Not Involved in the Elevated PYO Production in topA-RM

**Figure 11:**
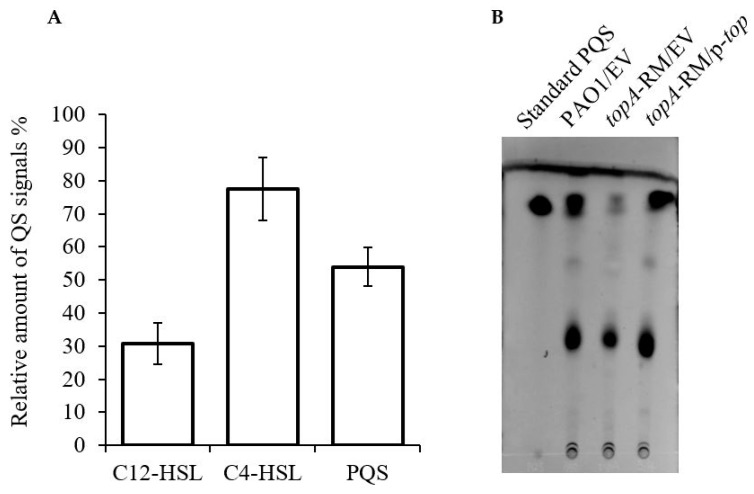
Relative amount of three QS signals in *topA*-RM compared with those in PAO1. (**A**) The relative amount of three signals in the culture supernatants of the two strains were measured using reporter systems. The relative percentage of signals was calculated by comparing the maximal cps values. The amount of each signal in PAO1 was designated as 100%. The means from three experiments are shown and the error bars represent standard deviations. (**B**) TCL analysis of PQS in the cultures of PAO1, *topA*-RM, and the complementation strain. PQS in the cultures was isolated by organic extraction and analyzed on TLC. Extract from 500 µL of culture equivalent was loaded onto the TLC plate. EV: Empty vector control. p-*topA: topA* complementation construct.

Evidences have showed that quorum sensing is a key regulatory system for the production of PYO in PAO1 [24]. Both the *rhl* system and the PQS system are required for PYO production [24,25,26,45]. To determine whether the QS systems were involved in the activation of PYO production in *topA*-RM, the major *P. aeruginosa* QS signals were measured as previously reported [46]. Surprisingly, the results obtained indicate that the *N*-(3-oxododecanoyl)-l-homoserine lactone (C12-HSL) did not increase in *topA*-RM, but decreased instead (Figure 11A). Similarly, both *N*-butanoyl-l-homoserine lactone (C4-HSL) and PQS signal were also reduced slightly in *topA*-RM. PQS production estimated by thin layer chromatography (TLC) confirmed the reduction in *topA*-RM (Figure 11B). These results demonstrate that these QS signals were not involved in the increased production of PYO in *topA*-RM. The decrease of QS molecules suggests that the *prtN*-mediated induction of PYO was able to override the QS-mediated regulation.

## 3. Discussion

DNA topoisomerases can be divided into two classes (type I and type II) which can be further subdivided into type IA, IB, IC, IIA, and IIB based on structural, mechanistical, and evolutionary considerations [1]. In *P. aeruginosa topA* encodes the only type I topoisomerases, while the rest of the topoisomerases, *gyrA*, *gyrB*, *parC,* and *parE* encode enzymes belonging to the type II topoisomerases. This is in contrast to *E. coli* where there are two type I topoisomerases, topoisomerase I (Topo I), encoded by *topA*, and topoisomerase III (Topo III), encoded by *topB* [33]. Transposon insertion or deletion mutants in the *topA* gene coding for topoisomerase I could be isolated from *E. coli*, *Salmonella typhimurium* and *Shigella flexneri* [47,48,49,50]. Our results demonstrate that topoisomerase I in *P. aeruginosa* is essential for cell viability, but the topoisomerase I lacking the 13 residues at the C-terminus or the fragmented *topA* in *topA*-RM retained activity that appears to be sufficient for the viability of this microorganism under the laboratory conditions. Such an essentiality of topoisomerase I is also observed in *Mycobacterium smegmatis* [35], *Helicobacter pylori* [9] *Streptomyces coelicolor* [51], and *Mycobacterium tuberculosis* [52]. 

The fact that the transposon mutant and *topA*-RM could grow just as well as the wild type in laboratory conditions appears to contradict the essentiality of the topoisomerase I in *P. aeruginosa*. The most plausible explanation is that the amino acid residues at the C-terminus of TopA (13 residues in the transposon mutant and 59 residues in the *topA*-RM mutant) are dispensable for cell viability. Consistent with our results, *topA* insertion mutants are present in both the PAO1 and PA14 transposon mutant libraries, and the transposon insertions in those mutants are also located in the 3′ end of *topA*, disrupting the C-terminal part of the translated TopA (http://beta.pseudomonas.com/). The *topA66* mutation in *E. coli* has a single nucleotide deletion, which causes an open reading frame shift losing the last 100 amino acid residues. The mutant grows well under normal laboratory conditions [37,53]. It has also been reported that the last 126 amino acid residues at the C-terminus of *E. coli* topoisomerase I are not required for the enzyme to perform DNA relaxation activity in vitro or in vivo [54]. However, our data indicate that, in *P. aeruginosa*, the lacking of the C-terminal residues did alter the DNA supercoiling status (Figure 5), despite no effect on cell viability being observed.

The phenotypical changes caused by the alteration of topoisomerase I in *topA*-RM include T3SS expression, phenazine production, antibiotic susceptibility, initial biofilm formation and swarming motility. While these changes are initially assumed to be a result of the changed DNA supercoiling, the detailed regulatory mechanisms for the genes associated with these phenotypes actually differ and the effect on T3SS seems not to be related with supercoiling.

It has been shown that a homeostasis of DNA supercoiling is maintained mainly via the regulation of transcription of topoisomerase genes [3,6]. *topA*, encoding DNA topoisomerase I, is stimulated by DNA negative supercoiling, while the *gyrA*, encoding DNA topoisomerase II, is down-regulated by DNA negative supercoiling in *E. coli* [3,42,55,56]. A similar control mechanism has been described in *Mycobacterium* spp. [55]. In many prokaryotic species, environmental signals can be transduced to the cell through changes of DNA supercoiling, which renders gene expression and subsequent changed cellular processes. In other words, through perturbing the chromosome supercoiling various bacterial cellular processes are influenced by environmental conditions [3,4,57,58]. Hence, topoisomerase I, which is involved in supercoiling, can lead to altered gene expression and changes in multiple phenotypes [35,37,50,59,60,61]. 

Our results show that the promoter activity of *topA* was up-regulated in *topA*-RM, while the *gyrA* promoter activity was slightly down-regulated (Figure 6C,D). Being the sole topoisomerase I in *P. aeruginosa*, TopA may serve as a sensor of supercoiling in the cell and respond to any changes to maintain the homeostasis of supercoiling. This is supported by the observation that the expression of *algD* operon, previously reported to be regulated by supercoiling [62], was also elevated significantly in *topA*-RM (Figure S8). The *algD* operon encodes the majority of the enzymes required for alginate production, which affects *P. aeruginosa* PAO1 biofilm development and biofilm architecture [63]. In agreement with this observation, initial biofilm formation was significantly enhanced in *topA*-RM, as indicated in borosilicate tube assays while swarming motility in *topA*-RM was reduced (Figure 5).

There is currently no report linking *topA* with T3SS expression in any bacteria. The results in our study show that the alteration of TopA in both the transposon mutant and *topA*-RM resulted in transcriptional inhibition of the expression of T3SS genes and reduced T3SS effectors production. We explored two potential mechanisms for such a regulatory effect of on T3SS. The first potential mechanism was the involvement of the increased supercoiling caused by the impaired TopA. It is known that gene expression could be influenced by altered supercoiling [4], and indeed, the increased supercoiling in the wild type strain was rendered by the increasing NaCl concentrations in the culture media. However, increased osmotic pressure and the resulting elevated supercoiling did not repress T3SS expression as expected. T3SS expression was instead activated in the wild type under such conditions. Moreover, in the mutant *topA*-RM, despite the increased supercoiling as in the wild type PAO1 with increasing NaCl concentration, T3SS expression remained unchanged. These results effectively disapprove a direct role of DNA supercoiling in affecting T3SS expression. It seems more plausible that the TopA activity is, instead, correlated with T3SS expression. The impaired TopA in *topA*-RM may account for the decreased T3SS expression. Considering the importance of maintaining a homeostasis of supercoiling in bacteria and the role of TopA in this, it was no surprise to observe increased *topA* expression in *topA*-RM where supercoiling was significantly elevated compared with the wild type (Figure 6C). The activated *topA* caused by increased supercoiling under increased NaCl concentrations may explain the increased T3SS expression in the wild type under such conditions. The inability of T3SS activation in *topA*-RM under increased NaCl concentrations could be explained by the impairment of the TopA in this strain. Supporting this notion, the overexpression of wild type *topA* on a plasmid also increased T3SS expression, while the mutant *topA* could not affect T3SS expression levels. It is most likely that an intact TopA is required for full T3SS expression in *P. aeruginosa*. These results seem to suggest that TopA has other activity that does not relate to DNA supercoiling. It has been reported that Type IA topoisomerases from all domains of life also have RNA topoisomerase activity for RNA, whereas direct interaction between RNA polymerase and the zinc ribbon domains of DNA topoisomerase I has been observed in *Escherichia coli* [64,65]. Further studies are required to determine potential additional activities of the TopA in *P. aeruginosa*. 

We explored the involvement of the known T3SS repressor PtrB in the changed T3SS expression caused by impaired TopA. It is known that PtrB represses T3SS, and *ptrB* is subject to the negative regulation of another transcriptional regulator PrtR [66]. As the expression of *recA* is activated in *topA*-RM where TopA activity is partially impaired, the increased RecA activity may cause the cleavage of PrtR [66], the repressor of PrtN and PtrB. We speculate that the de-repression of *ptrB* and *prtN* is probably the respective cause of the decreased T3SS expression and activated expression of genes involved in the synthesis of bacteriocin pyocin [44]. The altered TopA in *topA*-RM likely caused impairment in DNA replication or recombination, which triggered the activation of *recA*. Our data demonstrate that, compared with that in PAO1, *recA* expression was indeed increased in *topA*-RM (Figure 8A), and the transcription activity of *ptrB* was up-regulated in *topA*-RM (Figure 8B). However, when we deleted *ptrB* in *topA*-RM, no changes in T3SS expression were observed. Therefore, in spite of the de-repression of *ptrB* in *topA*-RM, possibly caused by decreased availability of PrtR, *ptrB* was not the cause of the reduced T3SS expression observed in the mutant. The impairment of TopA probably played a dominant role in the changes in T3SS.

Changes of antibiotic susceptibility were also observed in *topA*-RM. The increased susceptibility to the broad-spectrum antibiotic ciprofloxacin in *topA*-RM may have to do with the fact that the fluoroquinolone functions by inhibiting DNA gyrase [67,68]. The impaired TopA could have enhanced the effect of ciprofloxacin on DNA topology. In addition, it has been reported that the protein–protein interaction between topoisomerase I and RNA polymerase plays an important role during bacterial stress responses including response to antibiotic treatment [64,69]. The increased susceptibility to rifampicin, streptomycin, and tobramycin could be a result of changed interactions between topoisomerase I and RNA polymerase in *topA*-RM. This seems to be in agreement with the observation of increased sensitivity to trimethoprim and quinolone antimicrobials in *E. coli topA66* mutant [37]. Reduced topoisomerase I-RNA polymerase interaction and impaired SOS response are believed to be responsible for such changes [37]. 

One of the most obvious changes in *topA*-RM was the change of colony color. The *topA*-RM mutant had elevated phenazine production, resulting in more green color than the wild type (Figure 10). Copious PYO was produced in the mutant as confirmed by HPLC (Figure S4). The increased PYO production was apparently caused by the significantly increased expression of *phz* related genes (*phzA1*, *phzA2*, *phzM,* and *phzS*) in *topA*-RM (Figure 3). As phenazine synthesis is under the control of QS in *P. aeruginosa*, it came as a surprise that the QS gene expression and the level of QS signal molecules were either unchanged or decreased in *topA*-RM. It was observed serendipitously during the T3SS investigation that the deletion of *prtN* in *topA*-RM background made the strain to have the normal color as PAO1, reverting the increased green color of the *topA*-RM strain. The elevated PYO production in *topA*-RM clearly required the increased activity of PrtN. Because *prtN*, similar to *ptrB*, is also under the negative control of PrtR, the increased activity of PrtN is, in turn, a result of the decrease of PrtR by activated RecA in *topA*-RM. Future study is needed to determine the details of the interaction between PrtN and *phz* genes. Nevertheless, the results in this study not only point to a unique QS-independent regulatory pathway for phenazine production in *P. aeruginosa*, but also identify a mechanism that connects T3SS and PYO production through the transcriptional regulator PrtR. The connection between T3SS and phenazine production perhaps reflects the fact that both systems are involved in environmental adaptation and pathogenesis [70,71]. The pathway linking DNA supercoiling status and a key enzyme involved to these important phenotypes may represent a novel and underexplored bacterial regulatory pathway that could directly convey environmental cues such as osmotic stress. 

In summary, our study demonstrates the DNA topoisomerase I was essential in *P. aeruginosa*, and the partially impaired TopA had significant effect on multiple phenotypes including susceptibility to antibiotics and several important traits that are associated with pathogenicity. We have identified a QS-independent regulatory pathway of phenazine production that requires PrtN, and revealed that an intact TopA is required for full T3SS expression in *P. aeruginosa*. Such information provides new insights on the function of topoisomerases and points to an important role of TopA in regulating antibiotic resistance and virulence in *P. aeruginosa*.

## 4. Materials and Methods

### 4.1. Stains, Plasmids and Growth Conditions

The bacterial strains and plasmids used in this study are listed in Table S2. All strains were grown at 37 °C on an orbital shaker at 200 rpm unless specified otherwise. Antibiotics used in this study were all purchased from MP Biomedicals (Shanghai, China). Chemical agent (such as MgCl_2_, NaCl etc.) used in this study were all purchased from Tianli Chemical Reagent (Tianjin, China). Peptone and Yeast extract were purchased from OXOID (Hampshire, UK). For culture media, LB (Luria–Bertani) broth, Pseudomonas broth (PB) (2% Peptone, 0.14% MgCl_2_, 1% K_2_SO_4_) [66] and *Pseudomonas* Isolation Agar (PIA, Beijing Land Bridge Tech., Ltd., China) were used. PB is a medium to maximize pyocyanin production in liquid culture. Antibiotic concentrations used in this study as follows: For *P. aeruginosa*, 500 µg/mL carbenicillin (Car), 300 μg/mL tetracycline (Tet), 150 μg/mL gentamicin (Gen), and 300 μg/mL trimethoprim (Tmp); and for *E. coli*, 100 μg/mL ampicillin (Amp), 15 μg/mL Tet, 15 μg/mL Gen and 50 μg/mL kanamycin (Kan).

### 4.2. Construction of Mutant Strains

For construction of gene knockout mutants, the previously described *sacB*-based strategy was employed [32]. The DNA regions of the target genes were PCR amplified using primers listed in Table S1. Restriction sites were incorporated into the primers to facilitate cloning. The PCR products were digested with restriction enzymes and then cloned into pEX18Tc or pEX18Amp. For example, to construct the *ptrB* unmarked knockout mutant (*ΔptrB*), two fragments including upstream (1030 bp) and downstream (960 bp) for the intended deletion were amplified using two paired primers (*ptrB*-up1/*ptrB*-down1 and *ptrB*-up2/*ptrB*-down2). The PCR products were digested and then cloned into EcoRI/HindIII-digested pEX18Tc, yielding pEX18Tc-*ptrB*. The *ΔptrB* was obtained by using the triparental mating procedure in which the strain carrying the helper plasmid pRK2013 [72] was used together with the donor and recipient. The right mutant was further confirmed by PCR. The *ΔprtN* was generated as the same strategy for deletion of *ptrB* gene in PAO1 and the primers (*prtN*-up1/*prtN*-down1 and *prtN*-up2/*prtN*-down2) were used. For the *topA*-RM mutant, a 1.5-kb DNA fragment containing a part of *topA* was amplified by PCR using the primers P1 and P2 and then cloned into pEX18Amp, which was digested by SalI and EcoRI. The resulting plasmid was designated pEX18Amp-*topA*. A 4.238-kb fragment of pZ1918 [73] containing the *lacZ*, together with the Gen resistance (Gen^R^) determinant was ligated into BamHI-digested pEX18Amp-*topA* to create pEX18Amp-*topA*:*lacZ*Gm. It was then transferred into *P. aeruginosa* by triparental mating with the helper plasmid pRK2013 and selected on LB containing carbenicillin. To construct a *topA* conditional mutant, two PCR fragments containing upstream and downstream sequences of *topA* were ligated into pEX18Tc, which were amplified by two paired primers (P1/*topA*-down1 and *topA*-up2/*topA*-down2), as shown in Table S1 (due to the same antibiotic resistance marker between *topA*-RM and pUM108, we choose the pEX18Tc to construct the conditional mutant). Using the triparental mating method, the first recombination strain was obtained on PIA agar plates containing 300 μg/mL Tet. The pUM108-*topA* constructed by ligating pUM108 with the PCP product amplified using the P0/P3 primers was then introduced into the first recombination strain. The conditional mutant was then screened from the first recombination strain carrying pUM108-*topA* using the *sacB*-based strategy. The right conditional mutant was confirmed by PCR using the primers P0 and the *topA*-up primer. The *topA*-up primer only binds to the chromosome, but not the complementation plasmid. 

For generating *topA*-RM*ΔptrB* and *topA*-RM*ΔprtN* double mutants, the *ptrB* or *prtN* deleted mutation was introduced into the *topA*-RM background. These resultant mutants were verified by PCR.

### 4.3. Construction of lux-Based Expression Reporter and the Measurement of Expression Levels

The plasmid pMS402 carrying a promoterless *luxCDABE* reporter gene cluster was used to construct promoter-*luxCDABE* reporter fusions (p-*lux* reporter system), as reported previously [74]. The promoter regions of target genes were amplified by PCR using a Phusion high-fidelity PCR kit with the primers (Table S1). The PCR products were digested with BamHI-XhoI or BamHI-SalI (only *gyrA*), then they were cloned into the BamHI-XhoI site upstream of the lux genes on pMS402. Using these *lux*-based reporters, all gene expressions in different strains were measured as counts per second (cps) of light production with a Victor2 multilabel counter (Wallac model 1450; Perkin-Elmer, USA). In brief, overnight cultures of the reporter strains were diluted to an optical density of 0.2 at 595 nm (OD_595_) and cultivated for three additional hours before being used as inoculants. The cultures were inoculated into parallel wells on a 96-well black plate with a transparent bottom. Fresh culture (5 µL) was inoculated into the wells containing a total of 95 µL medium plus other components, and the OD_595_ in the wells was approximately 0.05. Filter-sterilized mineral oil (70 µL) was added to prevent evaporation during the assay. Promoter activities were measured every 30 min for 24 h. Bacterial growth was monitored at the same time by measuring the OD_595_ in the Victor3 multilabel plate reader. Expression on solid media was measured by plating the reporter strains in LB agar and imaging in a LAS300 imaging system (Fuji Corp., Miyagi Prefecture, Japan). If no otherwise specified, Tmp was added into the media with 300 µg/mL final concentration for plasmid maintaining. All experiments were repeated at least three times. For the relative quantification of the reporter expression on the plates, the software named multi gauge (version 3.0) was used to measure the emission amount of chemiluminescence when read by LAS3000. It signifies the relative density value accumulated as linear data by a CCD camera in the image surface. AU stands for Arbitrary Unit. The final AU in the results was normalized by the area of the region of interest. 

### 4.4. Complementation of Mutants

The DNA regions covering the entire target genes were PCR-amplified. For complementation of *topA*, *ptrB* and *prtN*, the paired primers P1/P3, *ptrB*-sense/*ptrB*-antisense and *prtN*-up/*prtN*-down2 were used respectively (Table S1). The PCR products were digested with corresponding restriction enzymes, and then cloned into pUCP26. The resulting plasmids were then introduced into the *topA*-RM, *topA*-RM*ΔptrB,* or *topA*-RM*ΔprtN* by electroporation, respectively. The pUCP26 was also introduced into the mutants or PAO1 as a control. When TopA was overexpressed in PAO1, the PCR fragment with intact *topA* was ligated into the pUCP26 which was digested with the corresponding restriction enzymes and then introduced into the different strains. To construct the p-*topAΔ59* which was lacking the 59 C-terminal residues, the PCR fragments amplified using the paired primers P1/P3 were digested with BamHI and HindIII and the larger fragments were purified using the kit. Then, the p-*topAΔ59* was obtained by ligating the fragments into the pUCP26, which was digested with corresponding restriction enzymes.

### 4.5. Measurement of Pyocyanin Production

Pyocyanin was extracted from culture supernatants and quantified using previously reported methods with minor modifications [75]. Briefly, 9 mL chloroform was added to 15 mL culture supernatant. After extraction, the chloroform layer was transferred to a fresh tube and mixed with 3 mL 200 mM HCl. After centrifugation, the top layer (200 mM HCl) was removed and its A520 was measured. The amount of pyocyanin, in mg/mL, was calculated using the following formula: A520/A600 × 17.072 = μg of pyocyanin per mL. 

Pyocyanin was detected by HPLC, as previously described [76]. Briefly, overnight cultures were diluted to OD (600 nm) of 0.2 in 5 mL PB. Cultures were grown at 37 °C with shaking at 200 rpm nearly for 18 h. Samples were centrifuged at 20,000× *g* for 5 min, and the supernatant was filtered through a polytetrafluoroethylene membrane (pore size, 0.22 μm). A 60 μL sample was loaded on an analytical ZORBAX Eclipse XDB-C18 column (5 μm particle size, 4.6 × 250 mm^2^). HPLC-based separation was carried out using a gradient of water—0.01% TFA (solvent A) to acetonitrile—0.01% TFA (solvent B) at a flow rate of 1 mL/min using the following protocol: Linear gradient from 0% to 15% solvent B for 2 min, linear gradient from 15% to 83% solvent B for 12 min, linear gradient from 83% to 100% solvent B for 2 min, and finally a linear gradient to 0% solvent B for 4 min. The total method time was 20 min. The retention time for pyocyanin was around 6.5 min.

### 4.6. Measurement of QS Signals

The relative amount of QS signals was measured using different systems as follow. The lasI-dependent C12-HSL production was estimated using a reporter strain *Agrobacterium tumefaciens* A136 (pCF218) (pMV26) [77]. The C4-HSL signal was measured using the PDO100 strain (*rhlI* mutant) containing pKD-*rhlA*, which was developed by fusing the C4-HSL responsive *rhlA* promoter upstream of *luxCDABE* and then introduced the construct into PDO100 [46,78]. *Pseudomonas* Quinolone Signal (PQS), a third intercellular signal, was measured using a *pqsA* promoter-based *P. aeruginosa* strain. In the assay, overnight cultures of the reporter strains were diluted 1:300 in LB media, and 90 μL of these solutions was added to the wells of a 96-well black plate. Ten microliters of filter-sterilized culture supernatants, which were taken from late-exponential (for C12-HSL or C4-HSL) or stationary phase (for PQS), or LB media (as a control) was added to the wells, the luminescence (measured in cps) and OD_595_ values were measured every half hour for a total of 24 h using a Victor2 multilabel counter (Wallac model 1450; Perkin-Elmer, USA). The relative levels of QS signals were calculated as follows: (cps–cps of the control)/optical density (OD) values, indicating the approximate light output per cell. The relative percentage of signals was calculated by comparing the maximal cps values. The amount of each signal in PAO1 was designated as 100%.

PQS signal quantification was also determined by thin layer chromatography (TLC) as previous report [24]. Briefly, bacterial strains were grown in LB media until they reached stationary phase (approximate 12 h). Samples were centrifuged at 20,000× *g* for 5 min, and the supernatant was filtered through a polytetrafluoroethylene membrane (pore size, 0.22 μm). Aliquots of culture supernatants were subjected to two extractions by the addition of one volume of acidified ethyl acetate (0.01% acetic acid). The organic phase was transferred to a fresh tube and dried to completion. The solutes were re-suspended in methanol for TLC analysis. The sample was spotted onto TLC plate which had been previously soaked for 30 min in 5% KH_2_PO_4_ and activated at 100 °C for 1 h. Extracts (PQS) were separated using a dichloromethane: Methanol (95:5) system until the solvent front reached the top of the plate. The plate was visualized using a UV and photographed. Standard QS signals were used as a positive control.

### 4.7. Initial Biofilm Formation Assay

The initiation of biofilm formation was measured in a static system, as previously described [79], with minor modifications. Visualization of initial biofilm formation was carried out in 15 mL borosilicate tubes. Briefly, cells from overnight cultures were inoculated at 1:100 dilutions into LB media supplemented with appropriate antibiotics and grown at 37 °C for 16 h. Biofilms were stained with 0.1% crystal violet and tubes were rinsed three times to remove unbound dye by submerging the tubes in distilled water and then photos were taken. To quantify the biofilm formation, the remaining crystal violet was dissolved in 1 mL of 95% ethanol and the absorbance of 0.9 mL portion of this solution was measured at 600 nm.

### 4.8. Bacterial Motility Assay

Bacterial motilities were assessed as described previously with slight modifications [80]. The media used for the swarming motility assay consisted of 0.5% agar, 8 g/L nutrient broth mix, and 5 g/L glucose. The media used for the swimming motility assay was tryptone agar containing 10 g/L tryptone, 5 g/L NaCl and 0.3% agar. LB broth solidified with 1% agar was used for the twitching motility assay. For swarming and swimming motilities, bacteria were spot inoculated onto plates as 2 mL aliquots taken directly from overnight LB cultures. For twitching motility, strains were stab inoculated with a sharp toothpick to the bottom of the Petri dish from overnight LB cultures. After inoculation, swarm plates were incubated at 37 °C and swim plates at 30 °C for 12–14 h, and twitch plates were incubated at 37 °C for 24 h. Photographs were taken with the LAS-3000 imaging system. Quantification of swarming motility was performed by evaluating the diameter of the covered areas.

### 4.9. Chloroquine Gel Electrophoresis

The pUCP26 was introduced into each of the strains by electroporation to identify differences in topoisomers between strains [38]. Strains carrying pUCP26 (or pKD-*exoY*) were grown to mid-logarithmic phase in 10 mL of LB broth containing 100 µg/mL of Tet to maintain the plasmid (or 50 µg/mL of Kan for pKD-*exoY*) at 37 °C with shaking at 200 rpm (For pKD-*exoY*, 300 µg/mL of Tmp was added into the broth), 5 mL of culture was harvested, and plasmid DNA was purified with the BIOER plasmid mini-pre kit according to the manufacturer’s instructions. Plasmid DNA (500 ng of DNA in a 25 µL final volume) was separated by gel electrophoresis on 1% agarose gels containing 5 µg/mL of chloroquine for 5 h at 30 V. After electrophoresis, gels were immersed in deionized water for 3 h to remove chloroquine before being stained with ethidium bromide (2.5 µg/mL) for 1 h. After being briefly washed again with water, the gel was placed on an ultraviolet transilluminator, and images were captured. For different concentration of NaCl treatment, strains were grown in LB with 0% NaCl for overnight and aliquots of culture were subjected to treat with different concentration of NaCl for 3 h with agitation before the plasmids were isolated using the kit. The topoisomers were measured as above. 

### 4.10. Protein Secretion Measurement

Bacteria were grown in T3SS inducing conditions (LB containing 5 mg/mL EGTA and 20 mM MgCl_2_) for 6–7 h at 37 °C [28]. Samples were equalized to 1.5 (OD_600_) in 1 mL to ensure equal loading of protein. Following the removal of bacterial cells by centrifugation at 14,000× *g*, proteins in the 900 µL supernatant were precipitated following the procedure in reference [81]. The pellet was dissolved in protein loading buffer, boiled and subjected to separation by 12.5 % SDS-PAGE. Protein bands were visualized directly by Coomassie staining. Quantification was performed using GeneTools software (Syngene, Cambridge, UK).

### 4.11. Statistical Analysis

All statistical analyses were performed by GraphPad Prism version 5 (GraphPad Software, La Jolla, CA, USA). The two-tailed unpaired *t*-test was used to analyze the data. *P* values of less than 0.05 (*P* < 0.05) were considered significant (*) and *P* < 0.005 considered very significant (**). Values are expressed as mean ± SD.

## Supplementary Materials

Supplementary materials can be found at https://www.mdpi.com/1422-0067/20/5/1116/s1.
