# Supplementary material for: Regulatory Effect of DNA Topoisomerase I on T3SS Activity, Antibiotic Susceptibility and Quorum- Sensing-Independent Pyocyanin Synthesis in Pseudomonas aeruginosa
Source: Int J Mol Sci. 2019 Mar 5;20(5):1116. doi: 10.3390/ijms20051116 (PMC6429228; doi:10.3390/ijms20051116)
Supplement: Supplementary file 1 [file ijms-20-01116-s001.pdf]

International Journal of *Molecular Sciences*

MDPI

Article

Regulatory effect of DNA topoisomerase I on T3SS activity, antibiotic susceptibility and quorum-sensing-independent pyocyanin synthesis in *Pseudomonas aeruginosa*

Supplementary Materials

Table S1. Primers used in this study

Primer	Sequence (5'→3')	Restriction site	Usage in the study
prtN-up1	TAGGGATCCGGGAAGTTGTGGA ACTCA	BamHI	Paired with *prtN*-down1 for *prtN* deletion mutant construction
prtN-down1	TATGTCGACTGGCTGCATGGTAT TCCC	SalI	Paired with *prtN*-up1 for *prtN* deletion mutant construction
prtN-up2	CTAGTCGACACAGCATCGCATC CTGAG	SalI	Paired with *prtN*-down2 for *prtN* deletion mutant construction
prtN-down2	CTGAAGCTTTCCTTCGAGACCCT TGCC	HindIII	Paired with *prtN*-up2 for *prtN* deletion mutant construction
prtN-up	CATCTCGAGGCGGGTGAAGTTC GTCTA	XhoI	Paired with *prtN*-down for *prtN* reporter construction
prtN-down	TCTGGATCCTGCATGGCCTTGTG ACTA	BamHI	Paired with *prtN*-up for *prtN* reporter construction
recA-up	ATTCTCGAGTGCCAGTTCAACG GCGAC	XhoI	Paired with *recA*-down for *recA* reporter construction
recA-down	ACTGGATCCTGGTCTTGCCCGAG GATT	BamHI	Paired with *recA*-up for *recA* reporter construction
ptrB-up1	TGCGAATTCATGGGTGGTGGCG GTGTC	EcoRI	Paired with *ptrB*-down1 for Δ*ptrB* construction
ptrB-down1	ACTGGATCCTTCGTTGGCGTGAT CGGC	BamHI	Paired with *ptrB*-up1 for Δ*ptrB* construction
ptrB-up2	ATAGGATCCCCGTTGAACCGAC CTCAT	BamHI	Paired with *ptrB*-down2 for Δ*ptrB* construction
ptrB-down2	TTAAAGCTTACGCCACATCTCGC CGCT	HindIII	Paired with *ptrB*-up2 for Δ*ptrB* construction
topA- down1	ATATCTAGACGCCAACCTGATG GAC	XbaI	Paired with P1 for *topA* conditional mutant construction.
topA- up2	ATATCTAGAGCGAACGAGCCAA GGTCA	XbaI	Paired with *topA*-down2 for *topA* conditional mutant construction
topA- down2	TAGAAGCTTGAGCCTGAATCCG AGAGA	HindIII	Paired with *topA*-up2 for *topA* conditional mutant construction
gyrA-up	TTAGTCGACCCTGATGAGCCGC AAG	SalI	Paired with *gyrA*-down for *gyrA* reporter construction
gyrA-down	TTAGGATCCGTAGGGCTTGTTCC AGT	BamHI	Paired with *gyrA*-up for *gyrA* reporter construction
algD-up	TCACTCGAGCGTATCGTTCATCT GCGT	XhoI	Paired with *algD*-down for *algD* reporter construction
algD-down	ATTGGATCCTAACCCGACCTTGG ATGC	BamHI	Paired with *algD*-up for *algD* reporter construction
topA-up	TATCTCGAGCTTCGTCCTGGTGA GTC	XhoI	Paired with *topA*-down for *topA* reporter construction
topA-down	TGAGGATCCAGGTACTTGTTGAT GGTC	BamHI	Paired with *topA*-up for *topA* reporter construction
phzM-up	TGCCTCGAGAACACAGAACGCT CGTAC	XhoI	Paired with *phzM*-down for *phzM* reporter construction
phzM- down	TAAGGATCCACGTAGACGCAAC	BamHI	Paired with *phzM*-up for *phzM* reporter

	GGGAC		construction
phzS- up	ATTCTCGAGTTGGAGCCCATCTAACCG	XhoI	Paired with *phzS*-down for *phzS* reporter construction
phzS-down	TCAGGATCCACTGCGGATAGGCGTTGC	BamHI	Paired with *phzS*-up for *phzS* reporter construction
ptrB- sense	TCTAAGCTTTGCTTCAGGTACGGCGGC	HindIII	Paired with *ptrB*-antisense for *ptrB* complementation construction
ptrB- antisense	TGCGGATCCTCGTGTCTCCTTTCACTG	BamHI	Paired with *ptrB*-sense for *ptrB* complementation construction
ptrB-up	CGTCTCGAGGCTTGTGAATGGTGTTCTGC	XhoI	Paired with *ptrB*-down for *ptrB* reporter construction
ptrB-down	CATGGATCCTGAGGTCGGTTCAACGG	BamHI	Paired with *ptrB*-up for *ptrB* reporter construction
M13F-forward	TGTAAAACGACGGCCAGT		Paired with pUC-based plasmid for *topA*-RM genotype check
M13R-reverse	CAGGAAACAGCTATGACC		Paired with pUC-based plasmid for *topA*-RM genotype check
P0	TAGGTCGACGTCGATACTCACGGCAAC	SalI	Paired with P3 for complementation using pUM108
P1	CAGGAATTCGTCGATACTCACGGCAAC	EcoRI	*topA*-RM construction or genotype check
P2	ATAGTCGACCGCCAACCTGATGGAC	SalI	*topA*-RM construction or genotype check
P3	CTGAAGCTTGACCTTGGCTCGTTCG	HindIII	*topA*-RM genotype check or Paired with P0 for complementation using pUM108

Table S2. Strains and plasmids used in this study

Stains & Plasmids	Description	Source
Stains		
P. aeruginosa		
PAO1	Wild-type, laboratory strain	This lab
ΔprtN	PAO1 with an unmarked deletion of *prtN*	This study
topA-RM	The first recombination strain contains the three disrupted *topA* fragments and pEX18Amp on the chromosome Gen^R, Car^R	This study
topA-RM*ΔprtN*	PAO1 with an unmarked deletion of *prtN* on *topA*-RM Gen^R, Car^R	This study
topA-RM*ΔptrB*	PAO1 with an unmarked deletion of *ptrB* on *topA*-RM Gen^R, Car^R	This study
PDO100	*ΔrhlI*::Tn501 derivative of PAO1	[1]
topA-Tm	PAO1 transposon mutant of *topA*	This study
E. coli		
DH10B	*F-mcrA Δ(mrr-hsdRMS-mcrBC)80dlacZ ΔM15 ΔlacX74 deoR recA1 endA1 araD139 Δ(ara leu)7697 galU galKλ-rpsL nupG*	This lab
Agrobacterium tumefaciens A136 (pCF218) (pMV26)	HSL detection strain	[2]
Plasmid		
pEX18Amp	*oriT*+ *sacB*+ gene replacement vector with multiple-cloning site from pUC18; Amp^R	[3]
pEX18Tc	*oriT*+ *sacB*+ gene replacement vector with multiple-cloning site from pUC18; Tet^R	[3]
pRK2013	Helper vector; Tra^+, Kan^R	[4]
pUCP26	*E. coli-P. aeruginosa* shuttle cloning vector, Tet^R	[5]
pUM108	*E. coli-P. aeruginosa* shuttle cloning vector with unique sites include HindIII, SphI, PstI, SalI, AatII. Amp^R	[6]
pMS402	Expression reporter plasmid carrying the promoterless *luxCDABE* gene, Tmp^R, Kan^R	[7]
pZ1918-*lac*ZGm	Source plasmid of Gen^R cassette	[8]
pKD-*prtN*	pMS402 containing *prtN* promoter region	This study
pKD-*recA*	pMS402 containing *recA* promoter region	This study
pKD-*ptrB*	pMS402 containing *ptrB* promoter region	This study
pKD-*exoT*	pMS402 containing *exoT* promoter region	[7]
pKD-*exoS*	pMS402 containing *exoS* promoter region	[7]
pKD-*exoY*	pMS402 containing *exoY* promoter region	[7]
pKD-*algD*	pMS402 containing *algD* promoter region	This study
pKD-*topA*	pMS402 containing *topA* promoter region	This study
pKD-*gyrA*	pMS402 containing *gyrA* promoter region	This study
pKD-*pqsA*	pMS402 containing *pqsA* promoter region	[9]
pKD-*rhlA*	pMS402 containing *rhlA* promoter region	[7]

pKD-*phzA1*	pMS402 containing *phzA1* promoter region	This study
pKD-*phzA2*	pMS402 containing *phzA2* promoter region	This study
pKD-*phzM*	pMS402 containing *phzM* promoter region	This study
pKD-*phzS*	pMS402 containing *phzS* promoter region	This study
pKD-PA0985	pMS402 containing PA0985 promoter region	This study
pKD-PA0614	pMS402 containing PA0614 promoter region	This study
pKD-PA0636	pMS402 containing PA0636 promoter region	This study
pUM108-*topA*	*topA*, including the promoter, was ligated into pUM108, Amp^R	This study
p-*topA*	*topA*, including the promoter, was ligated into pUCP26, Tet^R	This study
p-*topAΔ59*	p-*topA*, lacking the 59 C-terminal residues, was ligated into pUCP26, Tet^R	This study
p-*prtN*	*prtN*, including the promoter, was ligated into pUCP26, Tet^R	This study
pEX18Tc-*ptrB*	Amp^R; ligation of a 1.5-kb EcoRI and SalI PCR fragment into the same site of pEX18Tc	This study
pEX18Amp-*topA*:*lacZ*Gm	Amp^R Gen^R; ligated the BamHI-digested *lacZ* fragment from pZ1918 into pEX-*topA*	This study

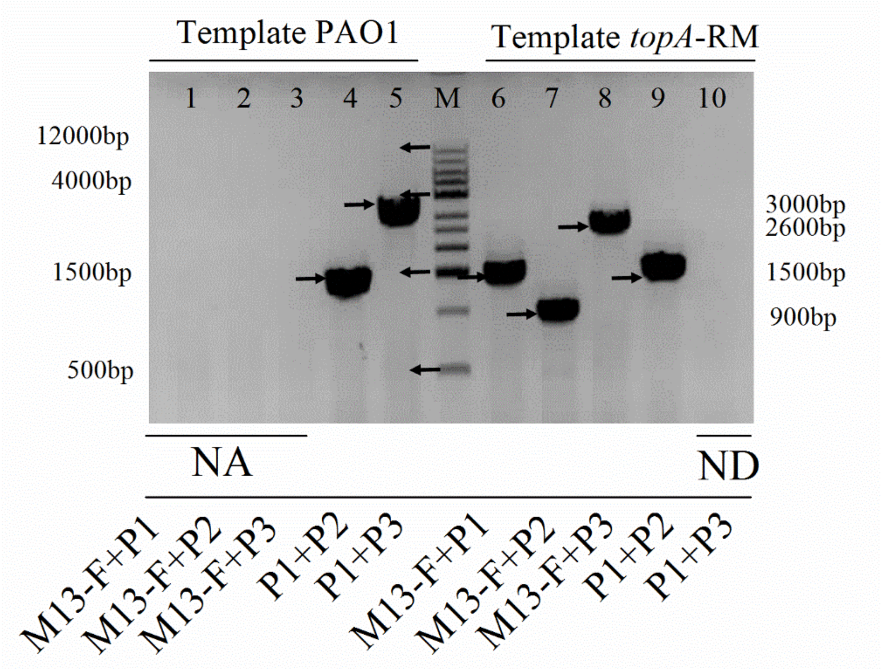

Figure S1. Verification of the genetic structure of *topA*-RM by PCR using specific primers. Different pairs of primers were used to amplify the specific fragments in *topA*-RM compared with the PAO1 (Genetic organization of *topA*-RM is shown in Figure 1A).

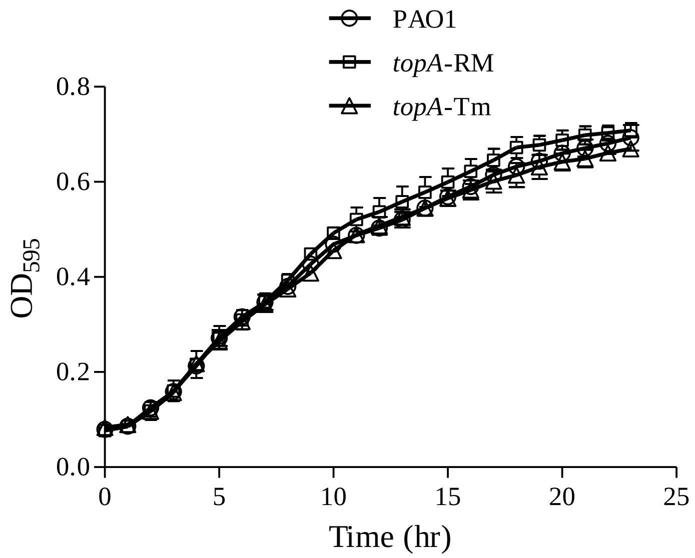

Figure S2. The growth of PAO1, *topA* transposon mutant and *topA*-RM. Overnight cultures of these strains were diluted 1:300 in LB, and 100 μl of these diluted cultures was added to the wells of a 96-well plate. OD_{595} values were measured every half hour. The means of triplicate experiments are shown, and the error bars indicate standard deviations. The results shown are representative of at least three independent experiments, all of which produced the similar results. *topA*-Tm: *topA* transposon mutant.

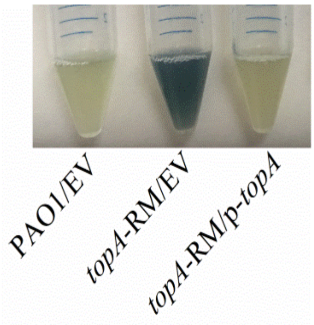

Figure S3. The enhanced pigmentation in *topA*-RM. PAO1, *topA*-RM, and the complementation strain were grown at 37°C with shaking at 200 rpm for 18h in PB medium. EV: empty vector control. p-*topA:* complementation construct with intact *topA* on pUCP26.

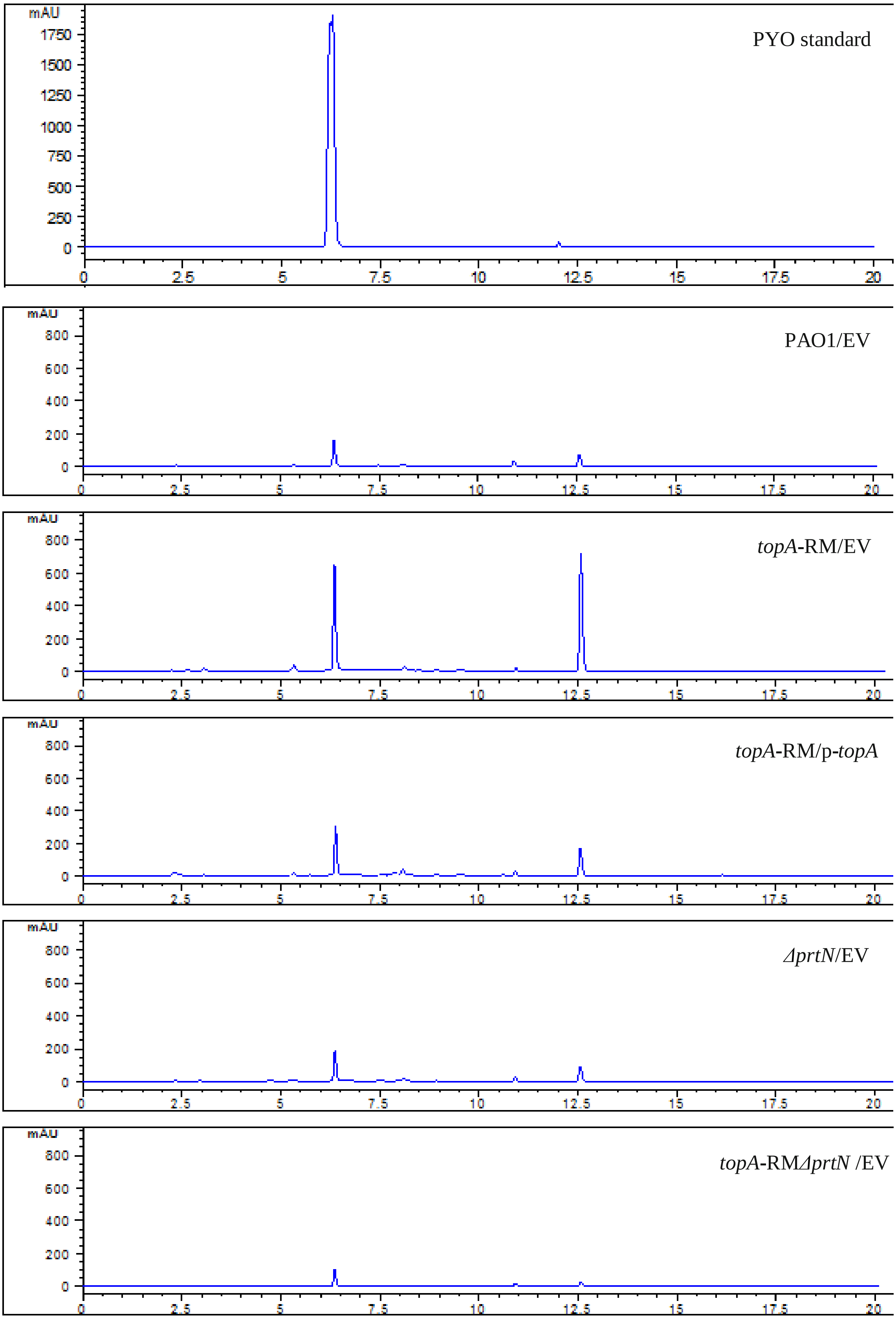
mAU
PYO standard
PAO1/EV
topA-RM/EV
topA-RM/p-topA
ΔprtN/EV
topA-RMΔprtN /EV

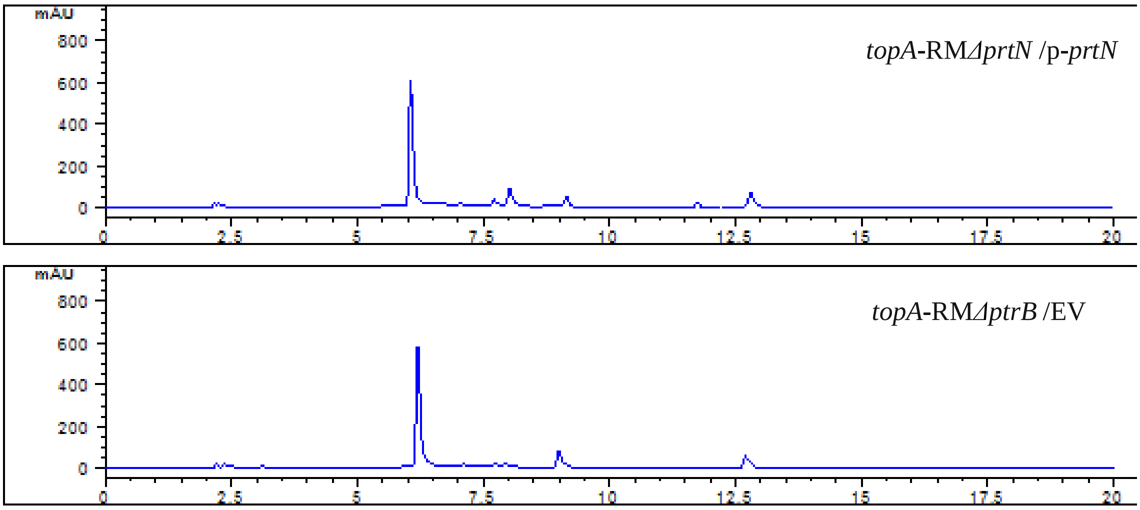

Figure S4. Pyocyanin production in different strains quantified by HPLC. The supernatants of culture samples were collected and filtered through a polytetrafluoroethylene membrane (pore size, 0.22 μm). A 60 μl sample was analyzed using HPLC. The standard PYO was used as a positive control. The retention time for pyocyanin was around 6.5 min. The relative quantity of PYO was indicated by the height of the peak. EV: empty vector control. p-*topA* and p-*prtN* indicate complementation strains containing an intact *topA* and *prtN* on pUCP26 respectively. The results shown are representatives of three independent experiments.

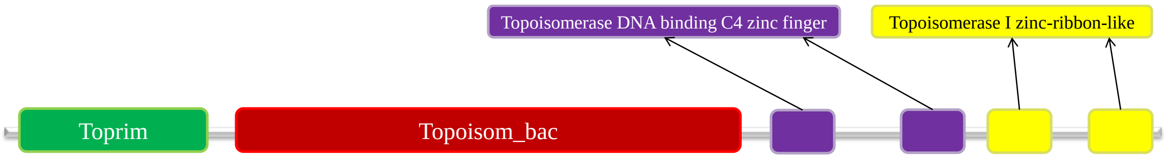


```
SEQUENCE       4  GKALVIVESPAKAKTINKYLGSDYVVKSSVGHIRDLPTSGSAAKKSADST   53
                  ||:|||||||||||||||||||.||||||:|||||||||||::.|...:.
PA3011         2  GKSLVIVESPAKAKTINKYLGSQYVVKSSIGHIRDLPTSGSSSSKEPAAK   51

SEQUENCE      54  STKTAKK-----PKKDERGALVNRMGVDPWHNWEAHYEVLPGKEKVVSEL   98
                  ..|:|.:     ||:..|..||:||||||.|.|:|.||:|||||||:.||
PA3011        52  GRKSASEAPALSPKEKARRQLVSRMGVDPEHGWKAKYEILPGKEKVIDEL  101

SEQUENCE      99  KQLAEKADHIYLATDLDREGEAIAWHLREVIGGDDARYSRVVFNEITKNA  148
                  ::||:.||.:|||||||||||||||||||.||||::||.|||||||||.|
PA3011       102  RRLAKDADTVYLATDLDREGEAIAWHLREAIGGDESRYKRVVFNEITKKA  151

SEQUENCE     149  IRQAFNKPGELNIDRVNAQQARRFMDRVVGYMVSPLLWKKIARGLSAGRV  198
                  |::||::||||:|:||||||||||:|||||||||||||:|||||||||||
PA3011       152  IQEAFSQPGELDINRVNAQQARRFLDRVVGYMVSPLLWQKIARGLSAGRV  201

SEQUENCE     199  QSVAVRLVVEREREIKAFVPEEFWEVDASTTTPSGEALALQVTHQNDKPF  248
                  |||||:|||||||||:||||||:|||.|...|..|..:..:||.:..:.|
PA3011       202  QSVAVKLVVEREREIRAFVPEEYWEVHADLGTAKGANVRFEVTREKGEAF  251

SEQUENCE     249  RPVNKEQTQAAVSLLEKARYSVLEREDKPTTSKPGAPFITSTLQQAASTR  298
                  :|:|:.|..||:..|:.:.|||.:|||:||:|:|.|||||||||||||.|
PA3011       252  KPLNEAQAMAALEKLKASAYSVAKREDRPTSSRPSAPFITSTLQQAASNR  301

SEQUENCE     299  LGFGVKKTMMMAQRLYEAGYITYMRTDSTNLSQDAVNMVRGYISDNFGKK  348
                  ||||||||||||||||||||||||||||||||.||:.||||:|.|.||:|
PA3011       302  LGFGVKKTMMMAQRLYEAGYITYMRTDSTNLSADAIGMVRGFIEDEFGQK  351

SEQUENCE     349  YLPESPNQYASKENSQEAHEAIRPSDVNVMAESLKDMEADAQKLYQLIWR  398
                  |||...|.|:|||.:|||||||||||||:....|..||.||::||.||||
PA3011       352  YLPGKANVYSSKEGAQEAHEAIRPSDVNLKPTQLSGMERDAERLYDLIWR  401

SEQUENCE     399  QFVACQMTPAKYDSTTLTVGAGDFRLKARGRILRFDGWTKVMPALRKGDE  448
                  ||||||||||:|.||:::|.||||.|:|:||||:|||:|:|:|...|..|
PA3011       402  QFVACQMTPAEYLSTSVSVTAGDFELRAKGRILKFDGYTRVLPQQSKPGE  451

SEQUENCE     449  DRILPAVNKGDALTLVELTPAQHFTKPPARFSEASLVKELEKRGIGRPST  498
```

```
                      |.:||.:.:|:.|.|::|.|:|||||||||:|||||||||||||||||||
PA3011            452 DDVLPEMKEGENLKLIKLDPSQHFTKPPARYSEASLVKELEKRGIGRPST     501

SEQUENCE          499 YASIISTIQDRGYVRVENRRFYAEKMGEIVTDRLEENFRELMNYDFTAQM     548
                      ||:||||||:||||...||||||||||:||||||.|:|..||:|.|||.|
PA3011            502 YAAIISTIQERGYVTTHNRRFYAEKMGDIVTDRLNESFANLMDYGFTAGM     551

SEQUENCE          549 ENSLDQVANHEAEWKAVLDHFFSDFTQQLDKAEKDPEEGGMRPNQMVLTS     598
                      |..||.||..|.:||.:||.|:.||.::|:.||..  |.|||.||..||:
PA3011            552 EEHLDDVAQGERDWKHLLDEFYGDFKKKLEVAEVS--EKGMRANQPTLTN     599

SEQUENCE          599 IDCPTCGRKMGIRTASTGVFLGCSGYALPPKERCKTTINLVPENEVLNVL     648
                      |.|..|||.|.||||||||||||||||:||||||||.|:||:|.:|:....
PA3011            600 IPCRECGRPMMIRTASTGVFLGCSGYSLPPKERCKATVNLIPGDEIAADD     649

SEQUENCE          649 EGEDAETNALRAKRRCPKCGTAMDSYLIDPKRKLHVCGNNPTCDGYEIEE     698
                      ||| :|:..||.|.|||.|.||||:||:|.|.|||:|||||.|.||||||
PA3011            650 EGE-SESRVLRGKHRCPICSTAMDAYLLDEKHKLHICGNNPDCPGYEIEE     698

SEQUENCE          699 GEFRIKGYDGPIVECEKCGSEMHLKMGRFGKYMACTNEECKNTRKILRNG     748
                      |::|||||:||.:||:||||||.||.|||||:..|||..||||||:|:||
PA3011            699 GQYRIKGYEGPSLECDKCGSEMQLKTGRFGKFFGCTNPTCKNTRKLLKNG     748

SEQUENCE          749 EVAPPKEDPVPLPELPCEKSDAYFVLRDGAAGVFLAANTFPKSRETRAPL     798
                      |.||||.|.:.:|||.|||.|..:||||||:|:||||:.|||:|||||||
PA3011            749 EAAPPKMDAIRMPELKCEKVDDIYVLRDGASGMFLAASQFPKNRETRAPL     798

SEQUENCE          799 VEELYRFRDRLPEKLRYLADAPQQDPEGNKTMVRFSRKTKQQYVSSEKDG     848
                      |.|:...:..|..|..||.||||:||:|...::|||||||:|||.||.||
PA3011            799 VSEIIPHKAELDPKYHYLCDAPQKDPDGRPAVIRFSRKTKEQYVQSEVDG      848

SEQUENCE          849 KATGWSAFYVDGKW-VEGKK     867
                      |.|||.|||..||| ||.|:
PA3011            849 KPTGWRAFYDGGKWKVEDKR      868
```

Figure S5. Sequence alignment of the topoisomerase I of *E. coli* and the TopA of *P. aeruginosa*. The amino acid sequences were retrieved from RCSB protein data bank (PDB: 4RUL) and *Pseudomonas* Genome DB and the alignment was performed with Pairwise Sequence Alignment (local alignment EMBOSS Matcher). The conserved domains are highlighted in different colors. Green: Toprim domain; Red: Topoisom_bac; Purple: Topoisomerase DNA binding C4 zinc finger; Yellow: Topoisomerase I zinc-ribbon-like domain. The 13 C-terminal residues missing in *topA*-Tm and the 59 C-terminal residues missing in *topA*-RM are boxed; the 13 residues are presented in green letters

and the rest of the 69 residue in red. Gap_penalty: 14; Length: 870; Identity: 587/870 (67.5%); Similarity: 701/870 (80.6%); Gaps: 9/870 (1.0%); Score: 3065.

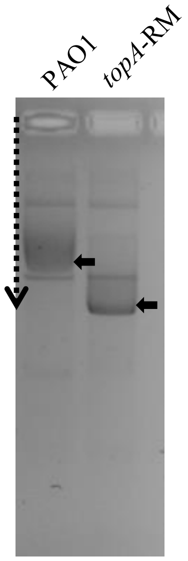

Figure S6. The state of DNA supercoiling in *topA*-RM and PAO1. DNA supercoiling was evaluated using the pKD-*exoY*, which was isolated from each strain and separated on 1% agarose gel containing 5 μg/ml chloroquine for 5 h at 30 V. Solid arrows indicate altered supercoiling. Dash arrows indicate the increased negative supercoiling. The result shown is representative of three independent experiments, all of which demonstrated the same trends.

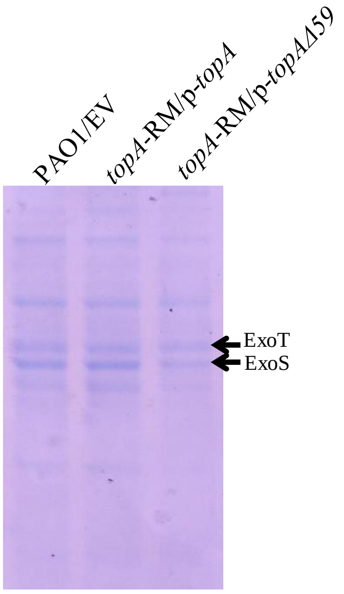

Figure S7. Secreted T3SS effects or protein levels in PAO1/EV, *topA*-RM/p-*topA* and *topA*-RM/p-*topAΔ59*. An intact *topA* and a truncated *topA* lacking the 59 C-terminal amino acid residues were introduced in *topA*-RM respectively to form the complementation constructs. The proteins in the culture supernatant of different strains were precipitated by TCA and analyzed by SDS-PAGE, followed by staining with Coomassie blue. ExoT and ExoS are indicated by solid arrows.

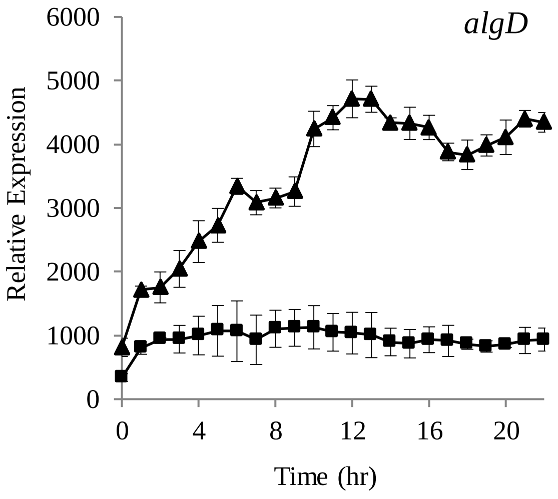

Figure S8. The *algD* promoter activity was up-regulated in *topA*-RM. The expression profiles were measured using p-*lux* reporter system every 30 min for 24 h at 37°C in LB. The expression values are presented as cps normalized to OD_{595}. The data in *topA*-RM are shown in triangle and those in PAO1 in square (data shown as averages from triplicate experiments ± standard errors of the means). The experiments were repeated at least three times. The result shown is representative of three independent experiments, all of which demonstrated the same trends.

References:

1. Brint, J.M.; Ohman, D.E. Synthesis of multiple exoproducts in *Pseudomonas aeruginosa* is under the control of RhlR-RhlI, another set of regulators in strain PAO1 with homology to the autoinducer-responsive LuxR-LuxI family. *J Bacteriol.* **1995**, *177*, 7155–7163.
2. Chambers, C.E.; Visser, M.B.; Schwab, U.; Sokol, P.A. Identification of N-acylhomoserine lactones in mucopurulent respiratory secretions from cystic fibrosis patients. *FEMS Microbiol Lett* **2005**, *244*, 297–304, doi:10.1016/j.femsle.2005.01.055.
3. Hoang, T.T.; Karkhoff-Schweizer, R.R.; Kutchma, A.J.; Schweizer, H.P. A broad-host-range Flp-FRT recombination system for site-specific excision of chromosomally-located DNA sequences: application for isolation of unmarked *Pseudomonas aeruginosa* mutants. *Gene* **1998**, *212*, 77–86.
4. Ditta, G.; Stanfield, S.; Corbin, D.; Helinski, D.R. Broad host range DNA cloning system for gram-negative bacteria: construction of a gene bank of *Rhizobium meliloti*. *Proc. Natl. Acad. Sci. USA* **1980**, *77*, 7347–7351.
5. West, S.E.; Schweizer, H.P.; Dall, C.; Sample, A.K.; Runyen-Janecky, L.J. Construction of improved *Escherichia-Pseudomonas* shuttle vectors derived from pUC18/19 and sequence of the region required for their replication in *Pseudomonas aeruginosa*. *Gene* **1994**, *148*, 81–86, doi:0378-1119(94)90237-2.
6. Chen, L.; Wang, W.; Sun, W.; Surette, M.; Duan, K. Characterization of a cryptic plasmid from *Pseudomonas* sp. and utilization of its temperature-sensitive derivatives for genetic manipulation. *Plasmid* **2010**, *64*, 110–117, doi:10.1016/j.plasmid.2010.05.003.
7. Duan, K.; Dammel, C.; Stein, J.; Rabin, H.; Surette, M.G. Modulation of *Pseudomonas aeruginosa* gene expression by host microflora through interspecies communication. *Mol.*

Microbiol. **2003**, *50*, 1477–1491.

8. Schweizer, H.P. Allelic exchange in *Pseudomonas aeruginosa* using novel ColE1-type vectors and a family of cassettes containing a portable *oriT* and the counter-selectable *Bacillus subtilis sacB* marker. *Mol. Microbiol.* **1992**, *6*, 1195–1204.
9. Liang, H.; Li, L.; Kong, W.; Shen, L.; Duan, K. Identification of a novel regulator of the quorum-sensing systems in *Pseudomonas aeruginosa*. *FEMS Microbiol Lett* **2009**, *293*, 196–204.
